# A Multi-Isotope Approach (δ^2^H, δ^18^O, δ^13^C, δ^15^N) for Discriminating Raspberry Production Systems and Assessing Agroecosystem Functioning

**DOI:** 10.3390/molecules31142459

**Published:** 2026-07-14

**Authors:** Roxana Elena Ionete, Diana Costinel, Ana Maria Simionescu, Marius Gheorghe Miricioiu, Augustina Pruteanu, Aura Irina Istrate, Oana Romina Botoran

**Affiliations:** 1 National Research and Development Institute for Cryogenic and Isotopic Technologies—ICSI Râmnicu Vâlcea, 4th Uzinei Street, P.O. Box Raureni 7, 240050 Râmnicu Vâlcea, Romania; roxana.ionete@icsi.ro (R.E.I.); diana.costinel@icsi.ro (D.C.); ana.simionescu@icsi.ro (A.M.S.); marius.miricioiu@icsi.ro (M.G.M.); 2 National Institute of Research—Development for Machines and Installations Designed for Agriculture and Food Industry—INMA, 013813 Bucharest, Romania; pruteanu@inma.ro; 3 Faculty of Biotechnical Systems Engineering, National University of Science and Technology Politehnica Bucharest, Splaiul Independentei 313, 060042 Bucharest, Romania; irina_aura.istrate@upb.ro

**Keywords:** stable isotopes, raspberry, agroecosystem functioning, hydroclimatic variability, sustainable agriculture

## Abstract

The development of sustainable and climate-resilient food systems increasingly relies on robust analytical methodologies capable of integrating environmental, biochemical, and management-related signals. In this study, a multi-isotope framework based on δ^2^H, δ^18^O, δ^13^C, and δ^15^N was applied to assess its capacity to discriminate between contrasting raspberry production systems and to provide chemically grounded indicators of agroecosystem functioning. Raspberry fruits (*Rubus idaeus* L.; cultivars *Opal* and *Delniwa*) were collected during the 2024–2025 growing seasons from two distinct systems in Romania: an organic open-field system and a rainfed agroforestry system. Stable isotope ratio analysis revealed system-dependent isotopic patterns, with the strongest differentiation observed for δ^15^N. Nitrogen isotope composition (δ^15^N) provided the strongest discrimination, with enriched values in organic fruits (2.73–9.77‰) and depleted values in agroforestry fruits (−3.01 to 0.62‰), reflecting differences in nitrogen sources and cycling pathways. Hydrogen and oxygen isotopes (δ^2^H: −60.46 to −4.62‰; δ^18^O: −6.19 to 10.41‰) were consistent with hydroclimatic variability and evaporative fractionation processes associated with soil–plant–atmosphere interactions. Carbon isotopes (δ^13^C: −28.14 to −22.62‰) provided complementary insights into plant water-use conditions. Multivariate statistical analysis supported the separation between production systems, while short-term fertilisation effects were secondary to system-level controls. The results suggest that raspberry fruits preserve an integrated isotopic fingerprint of production environment and management practices. From an analytical chemistry perspective, this work highlights the relevance of multi-isotope approaches as transferable tools for food authentication, traceability, and sustainability assessment, contributing to the broader application of stable isotope techniques across complex biological systems.

## 1. Introduction

Improving the sustainability of horticultural production requires indicators that can link management practices to underlying soil–plant–water interactions and nitrogen cycling. Organic agriculture and agroforestry are increasingly promoted because they can enhance ecosystem services such as soil quality, biodiversity conservation, and nutrient retention while potentially reducing reliance on synthetic inputs [1,2]. At the same time, intensive fertilisation has been associated with broader environmental pressures (e.g., acidification risks and reactive nitrogen losses), highlighting the need for monitoring approaches that are both field-relevant and mechanistically interpretable [3,4]. In this context, valorising agro-wastes into bio-based fertilisers aligns with circular bioeconomy goals and offers a practical route to restore soil fertility and support microbial functioning while reducing dependence on conventional mineral fertilisers [5,6].

In this broader context, the transition toward future food systems increasingly depends on strategies that combine climate resilience, sustainable resource use, and reliable approaches to product characterisation. Recent studies have highlighted that climate change is reshaping agrifood systems and exposing food supply chains to new vulnerabilities, while the valorisation of underused biomass, agricultural surpluses, and secondary biological resources is emerging as an important pathway toward more circular and sustainable food production [7,8,9]. At the same time, the development of value-added foods increasingly requires analytical tools capable of linking food composition to production environments and management conditions. For high-value horticultural products, such approaches are particularly relevant because they can strengthen traceability, support sustainability claims, and improve the monitoring of climate-sensitive production systems.

Berries are high-value foods with growing demand, and price differentials linked to geographical origin and production system increase vulnerability to mislabelling and fraud [10,11]. Raspberry (*Rubus idaeus* L.) is a perishable soft fruit whose quality and market value depend strongly on local environmental conditions and management, making it a relevant model for integrated agroecosystem assessment and supply chain transparency. Conventional traceability based on documentation is important but can be vulnerable to gaps; therefore, complementary analytical approaches that verify production claims directly from product composition are increasingly sought [10,12].

Stable isotope ratio analysis (SIRA) provides intrinsic “fingerprints” arising from climate, water sources, soil processes, and agricultural practices and has become a cornerstone in food authentication and provenance studies [10,13]. For plants, δ^2^H and δ^18^O primarily reflect the isotopic composition of source water and evaporative enrichment during transpiration, integrating local hydroclimate and water balance signals [14,15,16,17]. Carbon isotope composition (δ^13^C) records photosynthetic discrimination and is linked to stomatal regulation and intrinsic water-use efficiency, thereby reflecting plant physiological responses to water availability and management [18,19,20]. Nitrogen isotope composition (δ^15^N) reflects both the isotopic characteristics of nitrogen sources and fractionation processes associated with soil N transformations (e.g., mineralisation, nitrification, denitrification, and volatilisation) and has been widely applied to distinguish among fertilisation regimes, including organic and synthetic nutrient inputs [21,22,23].

In soft fruits, stable isotope applications remain less developed than in staple commodities, but evidence shows strong promise. A European survey of berries demonstrated that δ^13^C and δ^15^N (pulp) and δ^18^O (juice water) can discriminate geographical origin, while crop cover and fertilisation practices can measurably influence δ^18^O and δ^15^N and therefore must be considered when interpreting provenance signals [24]. However, Romanian datasets remain scarce beyond limited inclusion in wider European compilations, and there is limited field evidence on whether emerging bio-based fertilisers, particularly biochar–digestate formulations and organo-mineral biocomposites, leave a detectable short-term imprint on fruit isotope fingerprints during within-season application [5,6]. Addressing this gap is important both for mechanism-based interpretation (linking isotopes to soil–plant–water and N cycling) and for designing realistic traceability frameworks that account for management-driven variability.

Ensuring long-term food security requires agricultural systems that maintain productivity while reducing the environmental pressures associated with intensive farming. Over recent decades, the intensification of agricultural practices has contributed to soil degradation, nutrient imbalances, biodiversity loss, and broader ecosystem deterioration, raising concerns about the long-term sustainability of conventional production models [25,26]. In response, increasing attention has been directed toward alternative farming strategies and soil management solutions capable of reconciling crop productivity with environmental protection [27,28]. Among these, the development of bio-based fertilising materials derived from secondary biomass resources and agro-industrial residues represents a promising approach, as it supports nutrient recycling, improves soil functionality, and contributes to the transition toward more sustainable and circular agricultural systems.

Here, we evaluate a four-isotope fingerprint (δ^2^H, δ^18^O, δ^13^C, δ^15^N) of raspberry fruit across two locally managed production agroecosystems representing organic and agroforestry systems, using two cultivars (*Opal* and *Delniwa*) monitored over 2024–2025. We further test whether within-season application of bio-based fertilisers (biochar–digestate and organo-mineral biocomposite formulations) produces detectable shifts in fruit isotope ratios relative to the broader agroecosystem signal. Specifically, we hypothesise that (i) the production agroecosystem generates a distinct isotope signature, with δ^15^N reflecting contrasting N inputs and cycling and water isotopes reflecting differences in local water balance; (ii) interannual variability is detectable, particularly in water-related isotopes, consistent with year-to-year hydroclimatic shifts; and (iii) within-season fertilisation effects are smaller than the agroecosystem-level imprint. By linking isotopic patterns to soil–plant–water and nitrogen cycling processes, this study aims to support both agroecosystem monitoring and food traceability.

## 2. Results

### 2.1. Dataset Overview

Across the dataset, substantial variability was observed in water-related isotopes (Table 1; Table S1, Supplementary Material). Fruit water δ^2^H values ranged from −60.46 to −4.62‰, while δ^18^O values ranged from −6.19 to 10.41‰. Carbon isotope ratios (δ^13^C) fell within the expected range for C_3_ crops (−28.14 to −22.62‰) across production systems, cultivars, and years. Nitrogen isotope composition (δ^15^N) showed the clearest contrast between production agroecosystems. Fruit δ^15^N values ranged from −3.01 to 0.62‰ in the agroforestry agroecosystem and from 2.73 to 9.77‰ in the organic agroecosystem, with minimal overlap between systems. The relatively large standard deviations observed for δ^18^O and δ^2^H reflect the sensitivity of fruit-water isotopes to seasonal hydroclimatic variability. Because samples were collected throughout the harvest season under contrasting temperature and precipitation conditions, water-isotope compositions integrated both short-term meteorological fluctuations and longer-term evaporative effects, resulting in broader isotopic distributions than those observed for δ^13^C and δ^15^N.

### 2.2. Agroecosystem Differentiation in Fruit Isotope Composition

**Figure 1:**
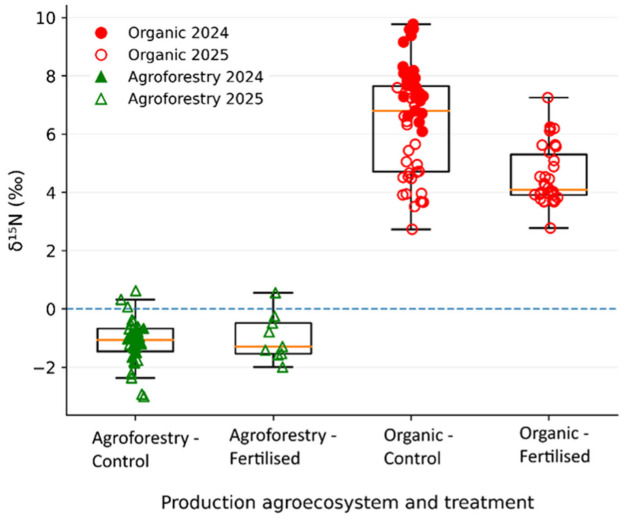
Distribution of raspberry fruit δ^15^N values (‰) across production agroecosystems and treatment categories. Boxplots summarise Control (unfertilised) and Fertilised samples. Individual observations are shown as symbols indicating production system and sampling year: red circles represent the organic agroecosystem and green triangles the agroforestry agroecosystem; filled symbols correspond to 2024 samples and open symbols to 2025 samples. The dashed horizontal line marks δ^15^N = 0‰, and the horizontal line inside each box indicates the median.

**Figure 2:**
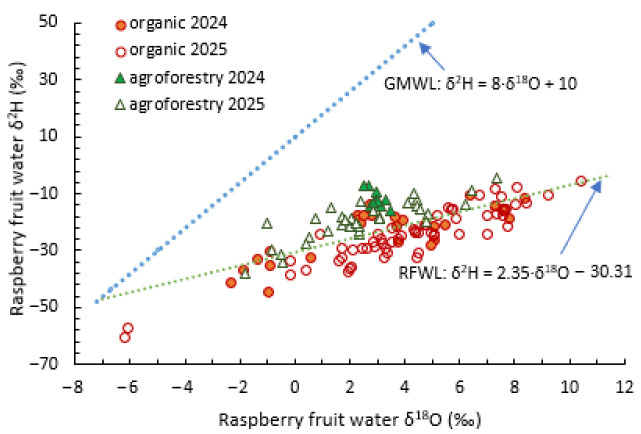
Fruit water isotopes (δ^2^H–δ^18^O) reflect hydroclimate and support agroecosystem separation.

The two production agroecosystems exhibited distinct isotope signatures across all analysed isotope systems (Table 1; Figure 1 and Figure 2). Nitrogen isotope composition (δ^15^N) provided the clearest separation, with organic fruits showing mean δ^15^N values of 5.70 ± 1.75‰ (*n* = 87), whereas agroforestry fruits were markedly depleted (−1.10 ± 0.81‰, *n* = 43) (Figure 1). This separation was highly significant (Mann–Whitney U test, *p* < 0.001) and persisted across years, cultivars, and fertilisation treatments.

Fruit water isotope compositions exhibited greater variability and were consistent with differences in agroecosystem context and hydroclimatic conditions. Across the full dataset, δ^18^O values ranged from −6.19 to 10.41‰, while δ^2^H values ranged from −60.46 to −4.62‰ (Table 1; Table S1, Supplementary Material). In dual-isotope space, fruit waters were evaluated relative to the Global Meteoric Water Line (GMWL) [14], which represents the theoretical isotopic relationship of global precipitation and provides a hydrological reference framework, as well as to the year-specific Local Meteoric Water Lines (LMWLs) derived from precipitation collected in the Vlădești/Vâlcea region during 2024 and 2025 (Figure 2 and Figure S3).

Fruit waters deviated markedly from the GMWL and aligned instead along an empirical Raspberry Fruit Water Line (RFWL) derived from the present dataset (δ^2^H = 2.35·δ^18^O − 30.31; R^2^ = 0.51) (Figure 2). The slope of the RFWL (2.35) was substantially lower than those of the GMWL (8.0), LMWL2024 (7.42) and LMWL2025 (6.72), consistent with substantial isotopic modification relative to meteoric source waters. Similar reductions in slope have been widely reported for evaporatively enriched plant and fruit waters and are generally attributed to isotopic fractionation occurring during soil evaporation, transpiration, and leaf–fruit water exchange processes [29,30,31,32]. Such reductions arise because preferential loss of lighter isotopes during evaporation progressively enriches the remaining water in ^18^O and ^2^H. Organic fruits generally occupied more enriched regions of δ^2^H–δ^18^O space and displayed broader isotopic ranges than agroforestry fruits.

Precipitation isotope datasets from the Vlădești/Vâlcea region provided additional hydrological context for interpreting fruit water isotope compositions (Figures S1–S4, Supplementary Material). Year-specific Local Meteoric Water Lines (LMWLs) were derived from precipitation isotope measurements collected during 2024 and 2025 and represent the most relevant meteoric baselines for evaluating isotopic modification of source water. Fruit waters consistently plotted below both the GMWL and the corresponding LMWLs and exhibited lower d-excess values than precipitation. Such deviations from meteoric water relationships are characteristic of evaporative enrichment because kinetic fractionation preferentially enriches residual water in the heavier isotopes, resulting in lower δ^2^H–δ^18^O slopes and reduced d-excess values [29,30,31,32]. Together, the reduced slope of the RFWL and the lower d-excess values support the interpretation that evaporative fractionation occurred during water transfer through the soil–plant–atmosphere continuum and fruit development. These patterns indicate that fruit water isotopes act as integrative indicators of plant–water relations and environmental conditions.

Carbon isotope composition (δ^13^C) exhibited a smaller but statistically significant difference between agroecosystems. Agroforestry fruits tended to be more depleted (−25.74 ± 0.88‰, *n* = 43) than organic fruits (−25.12 ± 1.08‰, *n* = 87), although substantial overlap was observed between the two groups. Statistical comparisons indicated significant differences between agroecosystems for all isotope systems (δ^15^N, *p* < 0.001; δ^2^H, *p* < 0.001; δ^18^O, *p* = 0.001; δ^13^C, *p* = 0.004; Table S3). Overall, δ^15^N contributed most strongly to agroecosystem discrimination, while fruit water isotopes and δ^13^C provided complementary information on environmental and plant-response variability associated with the contrasting production systems.

### 2.3. Interannual Variability and Hydroclimatic Context

Fruit isotope composition varied between the 2024 and 2025 growing seasons (Table 1). In the organic agroecosystem, mean fruit δ^15^N values decreased from 7.79 ± 1.01‰ (*n* = 26) in 2024 to 4.81 ± 1.21‰ (*n* = 61) in 2025, while mean δ^13^C values declined from −24.69 ± 1.28‰ to −25.31 ± 0.93‰. Statistical comparisons confirmed significant interannual differences for δ^15^N (*p* < 0.001) and δ^13^C (*p* = 0.008), whereas δ^18^O and δ^2^H did not differ significantly between years (Table S3).

In the agroforestry agroecosystem, δ^15^N values remained relatively stable (−1.14 ± 0.52‰ in 2024 and −1.08 ± 0.89‰ in 2025), whereas δ^13^C became more depleted, shifting from −24.83 ± 0.62‰ to −26.01 ± 0.76‰. Significant interannual differences were detected for δ^13^C (*p* < 0.001) and δ^2^H (*p* < 0.001), while δ^15^N and δ^18^O remained statistically unchanged (Table S3).

Climate records and precipitation isotope datasets from the Vlădești/Vâlcea region (Figures S1–S4) indicate substantial variability in temperature, precipitation, and meteoric water isotope composition between the two growing seasons. Such environmental differences are known to influence plant water relations and isotopic signatures and may therefore have contributed to the observed interannual variability, particularly for the fruit-water isotope systems. The observed shifts in δ^13^C are also consistent with changes in environmental conditions that can influence plant physiological responses, including stomatal conductance and water-use efficiency. Nevertheless, the isotopic separation between agroecosystems remained evident across years, indicating that agroecosystem-related differences were maintained despite seasonal variability.

### 2.4. Baseline Isotope Signatures in Control (Unfertilised) Fruits

Unfertilised control fruits provided the baseline isotope signatures against which fertiliser-related responses were evaluated (Table 1). The principal patterns observed in the full dataset remained evident when only control samples were considered, indicating that the production agroecosystem represented the dominant influence on fruit isotope composition.

Nitrogen isotope composition (δ^15^N) showed the strongest separation between agroecosystems. Organic control fruits consistently exhibited enriched δ^15^N values, ranging from 7.62 ± 1.04‰ and 7.96 ± 0.98‰ in 2024 to 4.80 ± 1.09‰ and 5.32 ± 1.66‰ in 2025 for *Delniwa* and *Opal*, respectively. In contrast, agroforestry control fruits remained centred on depleted signatures, ranging from −1.50 ± 0.38‰ to −0.78 ± 0.36‰ in 2024 and from −1.17 ± 1.20‰ to −1.04 ± 0.36‰ in 2025. Cultivar-related differences within each agroecosystem were small compared with the persistent system-level contrast. These results suggest that δ^15^N is associated with agroecosystem-specific differences in nitrogen dynamics that remained stable across cultivars and growing seasons.

Fruit water isotope compositions (δ^2^H and δ^18^O) and carbon isotope composition (δ^13^C) displayed greater overlap between agroecosystems but retained distinct baseline characteristics. Organic control fruits generally exhibited broader and more enriched δ^2^H–δ^18^O distributions, whereas agroforestry fruits tended to show narrower isotope ranges. Similarly, agroforestry control fruits were consistently more depleted in δ^13^C than organic controls, particularly in 2025. Although the magnitude of the δ^13^C difference was smaller than that observed for δ^15^N, the pattern was consistent across agroecosystems and may reflect differences in environmental conditions and plant physiological responses associated with the contrasting production settings. Together, these baseline signatures define the isotopic framework within which fertiliser-related responses can be interpreted and distinguish persistent agroecosystem effects from short-term management inputs.

### 2.5. Fertiliser-Related Isotope Responses Relative to Baseline

Fertiliser-related isotope responses were evaluated relative to the baseline signatures defined by unfertilised control fruits during the 2025 growing season (Table 1). Mann–Whitney U tests revealed no statistically significant differences between fertilised and unfertilised fruits for any isotope variable (all *p* > 0.05; Table S3). Across all isotope systems, fertilisation effects were therefore modest compared with the strong agroecosystem-specific signatures described above.

The most noticeable responses were observed for nitrogen isotope composition (δ^15^N). In the organic agroecosystem, fertilised fruits exhibited slightly lower mean δ^15^N values than their corresponding controls, with decreases of approximately 0.4–0.5‰ in both cultivars. For example, mean δ^15^N values decreased from 5.32 ± 1.66‰ to 4.82 ± 1.12‰ in *Opal* fruits and from 4.80 ± 1.09‰ to 4.43 ± 0.92‰ in *Delniwa* fruits. In the agroforestry agroecosystem, fertilised fruits remained centred on depleted δ^15^N signatures, ranging from −1.39 ± 0.50‰ in *Delniwa* to −0.16 ± 0.67‰ in *Opal* fruits. Despite these small shifts, fertilised and unfertilised fruits remained clearly within the isotope ranges characteristic of their respective production systems. These results suggest that short-term fertiliser application had a limited influence on the agroecosystem-specific nitrogen isotope signal observed in raspberry fruits.

Responses of fruit water isotopes (δ^2^H and δ^18^O) and carbon isotope composition (δ^13^C) were even less pronounced. In the organic agroecosystem, mean δ^18^O values changed only from 4.18 ± 3.78‰ to 4.45 ± 2.42‰ in *Opal* fruits and from 4.34 ± 3.67‰ to 4.33 ± 3.06‰ in *Delniwa* fruits following fertilisation. Similarly, mean δ^13^C values remained nearly unchanged, varying from −25.33 ± 1.03‰ to −25.27 ± 1.09‰ in *Opal* fruits and from −25.32 ± 0.81‰ to −25.31 ± 0.82‰ in *Delniwa* fruits. Comparable overlap between fertilised and unfertilised fruits was observed within the agroforestry agroecosystem.

Pearson correlation analysis (Table S2, Supplementary Material) revealed comparable relationships among δ^15^N, δ^13^C and fruit water isotopes in fertilised and unfertilised fruits. Together, these results indicate that short-term application of the bio-based fertilisers did not substantially alter either individual isotope signatures or the overall structure of the multi-isotope fingerprint. Under the conditions investigated here, agroecosystem-related differences remained considerably larger than the isotope shifts associated with short-term fertiliser application.

### 2.6. Multivariate Discrimination of Production Agroecosystems

To evaluate the combined discriminatory power of the four-isotope fingerprint (δ^2^H, δ^18^O, δ^13^C and δ^15^N), multivariate analyses were applied to all samples with complete isotope information across production agroecosystems and years.

**Figure 3:**
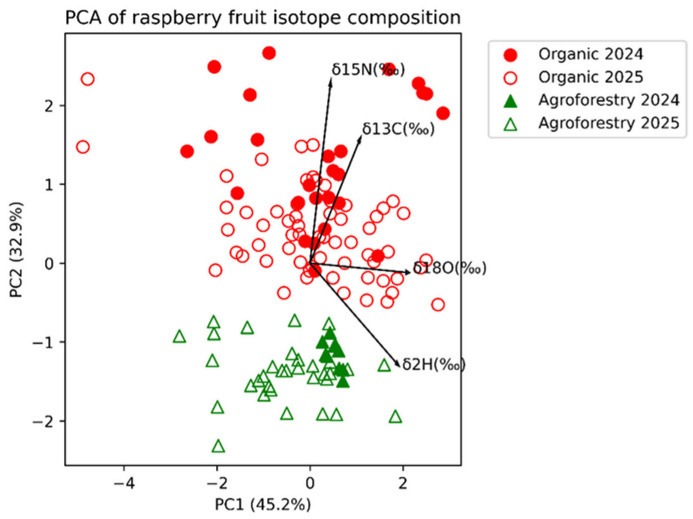
Principal component analysis (PCA) of raspberry fruit isotope composition based on δ^2^H, δ^18^O, δ^13^C and δ^15^N. PC1 and PC2 explain the percentages of total variance indicated on the axes. Arrows represent variable loadings of the four isotope tracers.

Principal component analysis (PCA) revealed structure in the isotope dataset (Figure 3). The first principal component (PC1, 45.2% of total variance) was influenced primarily by the water-isotope variables, particularly δ^18^O and, to a lesser extent, δ^2^H. The second principal component (PC2, 32.9%) was associated mainly with variation in δ^15^N and δ^13^C, with an opposing contribution from δ^2^H. Together, the first two components accounted for approximately 78.1% of the total variance.

Samples from the organic and agroforestry agroecosystems occupied largely distinct regions of PCA space, with differentiation occurring primarily along PC2. Organic samples generally exhibited higher PC2 scores, whereas agroforestry samples were associated with lower PC2 scores. This pattern is consistent with the strong contribution of δ^15^N to PC2 and supports the interpretation that variation in δ^15^N represented the principal contributor to agroecosystem discrimination within the multivariate dataset. The contribution of δ^13^C to PC2 and of the fruit-water isotopes to PC1 indicates that these tracers provided complementary information related to environmental and plant-response variability rather than acting as primary discriminators. Interannual variability was observable within both agroecosystems, with samples from the 2024 and 2025 growing seasons showing partial separation but substantial overlap. These patterns suggest that seasonal environmental variability contributed to isotopic variation, while agroecosystem-related differences remained detectable across years.

**Figure 4:**
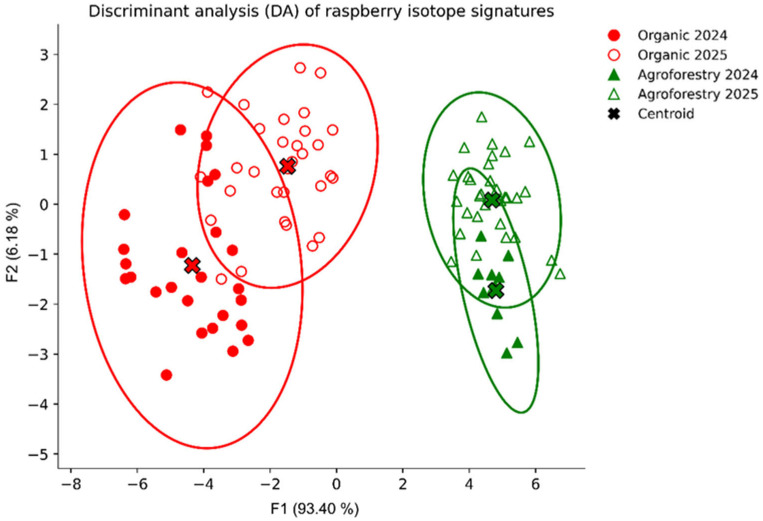
Discriminant analysis (DA) of raspberry fruit isotope signatures based on δ^2^H, δ^18^O, δ^13^C and δ^15^N. Ellipses illustrate the dispersion of samples within each agroecosystem–year group, and crosses denote group centroids.

Discriminant analysis (DA) also separated samples according to production system (Figure 4). In discriminant space, samples from the organic and agroforestry agroecosystems occupied largely distinct regions, although some overlap was observed among year-specific groups. Differentiation occurred primarily along the first discriminant axis (F1, 93.4% of explained discrimination), which was associated predominantly with variation in δ^15^N and, to a lesser extent, with contributions from the fruit-water isotope variables. This result is consistent with the strong agroecosystem-specific δ^15^N contrast observed throughout the dataset and is consistent with nitrogen isotope composition being the principal contributor to sample discrimination.

The second discriminant axis (F2, 6.2%) captured a comparatively small proportion of the total discrimination and was associated mainly with interannual variability within production systems. Accordingly, subsets of samples from the 2024 and 2025 growing seasons exhibited partial separation along F2 without affecting the strong discrimination between agroecosystems observed along F1. Ellipses and group centroids highlight the clustering of samples according to production system and sampling year.

Together, the DA results indicate that the combined isotope dataset effectively discriminated between the investigated agroecosystems, while also capturing secondary variability associated with seasonal environmental conditions. Although δ^15^N provided the dominant discriminatory signal, the remaining isotope variables contributed complementary information that improved characterisation of sample variability within and between agroecosystems.

Cultivar identity did not produce distinct clusters in either PCA or DA space, and fertilised fruits overlapped strongly with unfertilised controls within each agroecosystem. These patterns indicate that cultivar-related effects and short-term fertilisation responses were secondary to the dominant influence of agroecosystem context on fruit isotope composition.

Overall, the combined isotope dataset supported discrimination between the investigated agroecosystems. However, the multivariate analyses demonstrated that this separation was driven primarily by δ^15^N, whereas δ^2^H, δ^18^O and δ^13^C contributed complementary information associated with environmental variability and plant responses within the contrasting production systems. Together, these isotope tracers provided a more comprehensive characterisation of isotope variability associated with contrasting agroecosystems than could be achieved using a single isotope system alone.

## 3. Discussion

The combined use of nitrogen, water, and carbon isotopes revealed an isotopic fingerprint that differentiated the locally managed raspberry production agroecosystems. Among the tracers investigated, δ^15^N provided the strongest system-level separation and was consistent with persistent differences in agroecosystem-specific nitrogen dynamics. Fruit water isotopes (δ^2^H–δ^18^O) captured variability associated with hydroclimatic conditions and evaporative processes influencing the soil–plant–atmosphere continuum. Carbon isotopes (δ^13^C) contributed additional information that may reflect differences in environmental conditions and plant physiological responses associated with the contrasting production settings [19,29,33].

Importantly, the isotopic differences observed between production agroecosystems were larger and more persistent than the effects of short-term fertiliser application, indicating that fruit isotope composition integrates environmental and management influences operating over longer temporal and spatial scales. Although short-term management interventions may influence plant isotope composition, the present results suggest that agroecosystem-specific conditions exert a stronger control on the overall isotopic fingerprint of raspberry fruit.

Beyond their application for product traceability, the isotope patterns observed here highlight the potential of fruit isotope fingerprints as indicators of agroecosystem characteristics. By integrating information related to nitrogen dynamics, water relations, and environmental conditions, multi-isotope approaches provide a useful framework for evaluating production-system differences, sustainable management strategies, and agroecosystem resilience in horticultural systems. However, direct attribution of specific isotope signatures to individual biogeochemical processes or source pools would require complementary measurements of soil, water, and nutrient isotope compositions.

### 3.1. Nitrogen Isotope Composition as the Primary Agroecosystem Discriminator

Among the isotope systems investigated, δ^15^N provided the clearest and most interpretable separation between production agroecosystems. Fruits from the organic system consistently exhibited enriched δ^15^N values, whereas fruits from the agroforestry system clustered around depleted to near-zero signatures. The magnitude of this separation, together with its persistence across years, cultivars, and treatment categories, suggests that fruit δ^15^N reflects stable agroecosystem-specific differences in nitrogen dynamics.

Nitrogen isotope composition in plant tissues reflects the integrated influence of nitrogen source composition and fractionation associated with soil and plant processes, including mineralisation, nitrification, denitrification, ammonia volatilisation and plant uptake [21,23]. Consequently, plant δ^15^N values are widely recognised as integrative indicators of ecosystem nitrogen cycling rather than direct tracers of individual nitrogen sources [21,34]. Organic fertilisers derived from animal manures and composted organic materials typically exhibit enriched δ^15^N values, frequently ranging from approximately +5‰ to +25‰, whereas atmospheric N_2_ fixed biologically by symbiotic microorganisms generally produces nitrogen inputs centred close to 0‰ [22,23,35]. However, the isotopic composition of crops ultimately reflects the cumulative influence of source signatures and nitrogen transformations occurring within the soil–plant system.

Within this framework, the enriched δ^15^N signatures observed in fruits from the organic agroecosystem are consistent with a system characterised by long-term organic matter turnover and recycled nitrogen pools. In contrast, the depleted signatures observed in the agroforestry system are consistent with a distinct nitrogen cycling environment. Because isotopic measurements of soil nitrogen pools and fertiliser materials were not available, the present study does not permit direct attribution of the observed δ^15^N differences to specific nitrogen sources. Rather, the results support the existence of persistent agroecosystem-level differences in nitrogen dynamics.

The limited response of fruit δ^15^N to fertiliser application observed in the present study further supports this interpretation. Although small decreases in fruit δ^15^N values were detected following fertilisation, these changes remained minor relative to the approximately 6–7‰ separation between production systems. It is important to note, however, that the fertilisation experiment evaluated only short-term responses during a single growing season. Therefore, the present results indicate that fruit δ^15^N was influenced more strongly by pre-existing agroecosystem conditions than by short-term fertiliser additions, but they do not exclude the possibility that repeated or long-term management practices could progressively modify isotopic baselines over longer timescales.

Similar system-level contrasts in plant δ^15^N have been reported in studies aiming to differentiate agricultural production contexts, where long-term soil management history often exerts a stronger control on crop isotope composition than fertiliser type alone [23,35,36]. Consistent with this interpretation, δ^15^N contributed most strongly to sample separation in both principal component and discriminant analyses, highlighting its value as a robust indicator of agroecosystem identity and production context.

### 3.2. Fruit Water Isotopes as Indicators of Hydroclimatic and Microenvironmental Conditions

Fruit water isotopes (δ^2^H and δ^18^O) captured both agroecosystem differences and pronounced seasonal variability, reflecting their sensitivity to source-water composition and evaporative processes. In dual-isotope space, fruit water values deviated substantially from both the Global Meteoric Water Line (GMWL) and the year-specific Local Meteoric Water Lines (LMWLs), instead aligning along a lower-slope Raspberry Fruit Water Line (RFWL; δ^2^H = 2.35·δ^18^O − 30.31; R^2^ = 0.51). The slope of the RFWL (2.35) was markedly lower than those of the GMWL (8.0), LMWL2024 (7.42) and LMWL2025 (6.72), indicating substantial isotopic modification relative to meteoric source waters. Similar departures from meteoric water relationships have been widely documented in plant and fruit waters and are generally attributed to isotopic enrichment occurring during soil evaporation, transpiration, and leaf–fruit water exchange processes [29,30,31]. Comparable deviations from meteoric water lines have also been reported for apple fruit waters, where isotopic fractionation associated with evaporation resulted in fruit-water isotope compositions that diverged from local precipitation signatures and followed evaporation lines substantially different from meteoric water relationships [37].

The comparison with precipitation isotope datasets from the Vlădești/Vâlcea region provides additional support for this interpretation. Fruit waters consistently plotted below both the GMWL and the corresponding year-specific LMWLs and exhibited lower d-excess values than precipitation. These three observations—the markedly reduced slope of the RFWL relative to both local meteoric water lines, the displacement of fruit waters below the LMWLs, and the lower d-excess values—are recognised indicators of evaporative enrichment in ecohydrological studies of plant waters [29,30,31,32]. Such patterns arise because kinetic fractionation during evaporation preferentially removes lighter isotopes, progressively enriching the residual water in ^18^O and ^2^H while reducing both the δ^2^H–δ^18^O slope and d-excess values. Together, these observations support the interpretation that fruit-water isotope compositions were modified during water transfer through the soil–plant–atmosphere continuum and fruit development.

Although fruit water isotopes displayed greater within-group variability than δ^15^N, agroecosystem-related tendencies were still evident. Organic fruits generally occupied more enriched regions of δ^2^H–δ^18^O space and exhibited broader isotopic ranges than agroforestry fruits. These differences are consistent with contrasting microenvironmental conditions between production systems. Open-field cultivation exposes plants to higher radiation loads, greater evaporative demand, and larger fluctuations in soil moisture, whereas partial canopy cover in agroforestry environments can moderate temperature extremes, reduce vapour-pressure deficit, and influence soil water dynamics [1,32,33]. Such environmental differences may contribute to the distinct isotopic distributions observed between agroecosystems.

The pronounced interannual variability observed for fruit water isotopes further emphasises the importance of hydroclimatic forcing. Differences in precipitation amount, temperature, and meteoric isotope composition between 2024 and 2025 coincided with shifts in fruit δ^2^H and δ^18^O values. This interpretation is supported by the regional precipitation isotope datasets presented in the Supplementary Material and is consistent with previous studies demonstrating that fruit water isotopes integrate both climatic variability and local environmental conditions during fruit development [29,38,39].

Taken together, these results indicate that fruit water isotopes provide sensitive indicators of hydroclimatic and ecohydrological conditions operating within the soil–plant–atmosphere continuum. While they contributed less strongly than δ^15^N to agroecosystem discrimination, their responsiveness to source-water composition, evaporative enrichment, and local environmental variability provides important complementary information within the overall isotope framework.

### 3.3. Carbon Isotope Composition and Plant Physiological Responses

Carbon isotope composition (δ^13^C) showed a more moderate degree of agroecosystem separation than δ^15^N but nevertheless revealed consistent differences between production systems. Across the complete dataset, agroforestry fruits were slightly more depleted in ^13^C (−25.74 ± 0.88‰) than organic fruits (−25.12 ± 1.08‰), although substantial overlap occurred among individual samples. Although the numerical difference between agroecosystems was relatively small (approximately 0.6‰), it was consistent across the dataset and statistically significant (*p* = 0.004; Table S3). The substantial overlap between individual samples indicates that δ^13^C alone would not be sufficient for robust classification; however, the observed shift is consistent with the expected direction of isotopic responses to differences in canopy structure and microclimatic conditions. In contrast, cultivar-related differences within each agroecosystem were generally small, and fertilisation treatments produced only marginal shifts in fruit δ^13^C values.

In C_3_ plants, δ^13^C is widely interpreted as an integrative indicator of carbon isotope discrimination during photosynthesis, which is influenced by the balance between stomatal conductance and carbon assimilation [18,19,20]. Consequently, plant δ^13^C values are frequently used as indicators of environmental and physiological conditions affecting intrinsic water-use efficiency [19,33]. Lower (more negative) δ^13^C values are generally associated with greater discrimination against ^13^C, whereas less negative values may occur under conditions that restrict stomatal conductance and reduce carbon isotope discrimination.

The tendency toward more depleted δ^13^C values in the agroforestry agroecosystem is consistent with the microenvironmental conditions typically associated with partially shaded systems. Forest-edge canopy cover can reduce incoming radiation, moderate air temperature, decrease vapour-pressure deficit, and influence soil moisture dynamics relative to open-field conditions [1,32]. Such environmental differences may favour greater carbon isotope discrimination and could contribute to the more depleted δ^13^C values observed in agroforestry fruits. However, because physiological measurements such as stomatal conductance or water-use efficiency were not performed, this interpretation should be regarded as consistent with established isotope theory rather than direct evidence of the underlying mechanisms.

Interannual variability further supports the sensitivity of δ^13^C to environmental conditions. Significant differences between 2024 and 2025 were observed in both agroecosystems, with fruit δ^13^C values becoming more depleted in 2025. This pattern coincided with differences in seasonal hydroclimatic conditions and suggests that fruit δ^13^C integrates environmental influences operating during fruit development. Similar relationships have been reported for grapevine, apple, strawberry, and other perennial fruit crops, where carbon isotope discrimination responds to variation in water availability, canopy structure, and microclimate [33,40,41].

Although δ^13^C provided weaker discrimination between agroecosystems than δ^15^N, it contributed complementary information within the multi-isotope framework. Together with fruit water isotopes, δ^13^C helped characterise environmental variability and plant responses associated with contrasting production systems, thereby improving the overall interpretation of agroecosystem differences.

Therefore, δ^13^C should be interpreted primarily as a complementary indicator of environmental and physiological variability rather than as a direct tracer of production-system origin.

### 3.4. Interannual Variability and Ecological Context

Interannual hydroclimatic variability between the 2024 and 2025 growing seasons provides important context for interpreting the isotope patterns observed in raspberry fruits. Significant year-to-year differences were detected for several isotope variables, particularly δ^13^C and, depending on agroecosystem, δ^15^N and δ^2^H. Such variability is expected because stable isotope compositions in plant tissues integrate environmental conditions operating during fruit development, including precipitation regime, temperature, atmospheric demand, and source-water isotopic composition [29,38,42].

The strongest interannual responses were observed for fruit water isotopes, which are known to respond rapidly to changes in hydroclimatic conditions. The precipitation isotope datasets from the Vlădești/Vâlcea region (Figures S1–S4) indicate differences in both meteoric isotope composition and seasonal weather conditions between the two study years. These differences are consistent with the observed variability in fruit δ^2^H and δ^18^O values and support the interpretation that fruit water isotopes are sensitive indicators of environmental variability.

The reduced agroforestry fruit dataset obtained in 2024 also illustrates the challenges associated with rainfed production systems under variable climatic conditions. Rainfed perennial crops are often more sensitive to seasonal fluctuations in water availability than irrigated systems, particularly during critical stages of flowering, fruit set, and fruit development [43,44]. Consequently, climatic variability may influence not only isotope composition but also crop performance and fruit availability.

Field observations suggested greater vulnerability of the *Delniwa* cultivar in water-limited microsites, particularly in areas characterised by lighter soil texture and southern exposure. Although cultivar-related differences in isotope composition were generally small, these observations may help explain the reduced number of *Delniwa* samples available from the agroforestry system in 2024. Because these field observations were not directly quantified, they should be interpreted cautiously. Nevertheless, they are consistent with the importance of local soil and microclimatic heterogeneity in influencing cultivar performance within agroforestry environments [1,32].

Taken together, these observations highlight the importance of interpreting fruit isotope signatures within their broader environmental context. Hydroclimatic variability, local environmental conditions, and cultivar-specific responses can collectively influence both plant performance and isotope composition, particularly in low-input production systems where management interventions are intentionally limited.

### 3.5. Fertiliser Effects Relative to Baseline Isotope Signatures

Relative to the baseline signatures defined by unfertilised control fruits, fertiliser-related isotope responses were generally small and statistically non-significant across all isotope systems. Although slight shifts were observed for some treatment groups, particularly for δ^15^N in the organic agroecosystem, fertilised fruits largely remained within the isotopic ranges characteristic of their respective production systems. These findings indicate that short-term application of the tested bio-based fertilisers did not substantially modify the isotopic fingerprint of raspberry fruits during the study period.

The most noticeable treatment-related responses were observed for δ^15^N, where fertilised fruits exhibited slightly lower mean values than unfertilised controls in the organic agroecosystem. However, these changes were small relative to the approximately 6–7‰ separation observed between agroecosystems and did not alter the overall discrimination pattern. Similar observations have been reported in studies showing that plant δ^15^N often integrates nitrogen acquired from multiple soil and plant pools rather than reflecting solely recently applied fertiliser inputs [21,23,36].

The limited isotopic response to fertilisation is consistent with the integrative nature of isotope signals in perennial fruit crops. Developing raspberry fruits accumulate assimilates and nutrients over extended periods, during which newly introduced nitrogen may be mixed with pre-existing soil nitrogen reserves and internal plant nutrient pools. Consequently, short-term fertiliser additions may have a limited influence on fruit isotope composition relative to pre-existing soil–plant system conditions [21,34,45].

Responses of fruit water isotopes and carbon isotope composition were even less pronounced. The strong overlap observed between fertilised and unfertilised fruits suggests that environmental variability and agroecosystem-specific conditions exerted a greater influence on fruit δ^2^H, δ^18^O and δ^13^C values than the fertilisation treatments applied during a single growing season. Similar findings have been reported for perennial crops, where climatic and physiological factors often exert stronger controls on stable isotope composition than short-term management interventions [19,42].

It is important to emphasise that the present study evaluated only short-term responses to bio-based fertiliser applications. Therefore, the results should not be interpreted as evidence that fertilisation cannot influence fruit isotope composition. Rather, they indicate that under the conditions investigated here, short-term fertiliser inputs exerted a weaker influence on fruit isotope signatures than the pre-existing agroecosystem context. Long-term applications could progressively modify soil nutrient pools, nitrogen cycling processes, and associated isotopic baselines, potentially leading to stronger isotopic responses over time.

Overall, the persistence of agroecosystem-specific isotope signatures following fertilisation supports the use of isotope fingerprints as indicators of production context. At the same time, the results highlight the importance of distinguishing between short-term management effects and longer-term agroecosystem processes when interpreting stable isotope signatures in perennial fruit crops.

### 3.6. Implications for Food-System Resilience, Circularity, and Supply Chain Transparency

The multivariate analyses were generally consistent with the patterns observed for the individual isotope systems. Both principal component analysis and discriminant analysis differentiated samples from the organic and agroforestry agroecosystems, despite the relatively small geographical distance between the study sites and their shared regional climatic setting. The observed separation was associated primarily with variation in δ^15^N, while fruit water isotopes and δ^13^C provided complementary information. Together, these isotope tracers were associated with multiple aspects. Similar patterns were observed across years, cultivars, and fertilisation categories, suggesting that agroecosystem-related influences on isotopic composition remained detectable despite interannual variability.

These findings extend the relevance of stable isotope analysis beyond environmental characterisation by demonstrating that harvested raspberry fruits retain measurable isotopic differences associated with contrasting production systems. In the present study, isotope composition was more strongly associated with agroecosystem context than with short-term fertiliser inputs. The observed patterns are consistent with differences in nitrogen dynamics, water relations, and environmental conditions operating within the production systems, although direct attribution to specific biogeochemical processes would require complementary measurements of soil, water, and nutrient isotope pools.

From a food-systems perspective, the persistence of agroecosystem-specific isotope signatures has important implications for transparency and traceability. Stable isotope fingerprints can provide an intrinsic and independently verifiable record of production context, complementing documentary certification and conventional traceability approaches [13]. For high-value and highly perishable fruits such as raspberries, where production method, provenance, and sustainability claims may influence market value, isotope-based verification offers an additional layer of confidence for producers, retailers, and consumers.

The results are also relevant to ongoing discussions concerning resilience and circularity in food systems. Contemporary food-system research increasingly emphasises the need to evaluate not only agricultural productivity but also the capacity of production systems to maintain functionality under environmental variability while supporting efficient resource use and sustainable supply chains [46,47]. In this context, the consistent agroecosystem-related isotope patterns observed in fruit isotope composition suggest that isotope-based approaches may contribute to assessing how management practices and environmental conditions influence broader system characteristics over time.

Particularly noteworthy is the observation that short-term application of bio-based fertilisers produced only limited isotopic responses relative to the dominant agroecosystem signal. Although this does not diminish the agronomic or environmental value of such inputs, it suggests that their effects should be considered within the broader framework of long-term soil–plant–water interactions. This perspective aligns with circular bioeconomy strategies that seek not only to substitute conventional inputs but also to strengthen resource cycling, soil health, and agroecosystem resilience through sustained management practices [48,49,50].

At the same time, the present study addresses only one dimension of food-system sustainability: the biophysical characterisation of the production context. It does not directly assess productivity, economic performance, post-harvest losses, nutritional quality, or social dimensions of food security. Future research should therefore integrate multi-isotope approaches with agronomic, economic, and supply chain indicators in order to better connect field-scale ecological processes with broader food-system outcomes. Such integration would further strengthen the role of isotope-based tools in supporting sustainability assessment, supply chain transparency, and climate-resilient horticultural production systems.

Overall, the results suggest that isotope fingerprints can serve as useful indicators linking product composition to production-system characteristics. By connecting isotopic variation observed in agricultural products with information relevant to traceability, verification, and sustainability assessment, stable isotope approaches offer a promising framework for supporting resilient, transparent, and resource-efficient food systems.

## 4. Materials and Methods

### 4.1. Study Area and Locally Managed Production Agroecosystems

**Figure 5:**
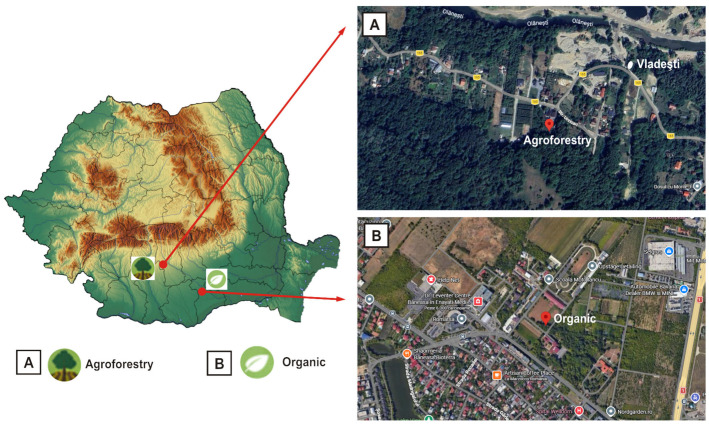
Location and landscape context of the two experimental raspberry plots in Romania. The red markers indicate the geographical positions of the study sites on the national map, and the red arrows link these locations to the corresponding satellite views. Blue markers identify the exact locations of the experimental plots: (**A**) agroforestry site near Vlădești, Vâlcea County (forest-edge context); (**B**) organic site at INMA Bucharest (peri-urban/open-field context). Source: Google Earth satellite imagery (https://earth.google.com/web/), accessed on 2 February 2026.

Raspberry fruits were collected from two locally managed production agroecosystems in Romania over two consecutive growing seasons (2024–2025): (i) an organic production plot at INMA, Bucharest (44°30′01″ N, 26°04′19″ E; hereafter referred to as “organic”) and (ii) an agroforestry-based plot at Vlădești, Vâlcea County (45°07′11″ N, 24°17′33″ E; hereafter referred to as “agroforestry”). Both plantations were established in mid-March 2023 using two raspberry cultivars (*Opal* and *Delniwa*) and covered approximately 1000 m^2^ at each site (Figure 5).

At INMA (Bucharest), the plantation comprised four rows (two per cultivar) arranged from the outset into three management zones: an unfertilised control and two fertiliser zones (fertiliser 1 (FER1) and fertiliser 2 (FER2)). Each zone extended approximately 26 m along the rows and was separated from adjacent zones by approximately 4 m access alleys that were maintained without fertiliser application. Fertilisers were applied only within the designated treatment zones. Row spacing was 3.3 m, and within-row spacing was cultivar-dependent (0.5 m for *Opal*; 0.75 m for *Delniwa*). Plants were trained on a trellis support system following standard Rubus horticultural practice (approximately 5–6 vigorous shoots per plant).

At Vlădești (Vâlcea), the plantation included six rows (three per cultivar) and followed the same three-zone layout (control, fertiliser 1 (FER1), and fertiliser 2 (FER2)). Each zone spanned approximately 34 m and was separated from adjacent zones by approximately 2 m access alleys that were maintained without fertiliser application. As at the INMA site, fertilisers were applied only within the designated treatment zones. Row spacing was 3.0 m, and within-row spacing was 0.5 m for both cultivars. In contrast to the INMA site, plants here were managed without trellis training in a more natural, agroforestry-edge setup. The plot is located adjacent to a forest edge with a multi-strata canopy (a tall overstory with dense understory vegetation), creating heterogeneous shading and wind shelter typical of an agroforestry microclimate.

The experiment was conducted under rainfed conditions at both sites. Given the separation distances between treatment zones, the absence of fertiliser application within the access alleys, and the lack of irrigation, potential lateral transfer of nutrients between adjacent treatment zones was considered limited, although it cannot be entirely excluded under field conditions.

Although the two experimental plots were of comparable size (approximately 1000 m^2^ each), the number of fruit samples obtained from the agroforestry system was lower than that obtained from the organic system. This difference reflects the management philosophy of the agroforestry site, where human intervention was intentionally minimised to preserve the natural development of the system. Consequently, fruit production was spatially heterogeneous, and some plants produced few or no harvestable fruits during the study period. This was particularly evident in portions of the plot located on sloping terrain adjacent to the forest edge, where periods of limited precipitation may have reduced water availability and contributed to lower fruit production. The resulting imbalance in sample numbers between agroecosystems was therefore a consequence of field conditions rather than differences in experimental plot size.

Key site characteristics and management descriptors for the two locally managed production agroecosystems are summarised in Table 2.

Baseline soil agrochemical properties (pH, humus content, available phosphorus, and available potassium) were determined by the Laboratory for Physico-Chemical Analyses of Soil Science, Agrochemistry and Environmental Protection of the National Research and Development Institute for Soil Science, Agrochemistry and Environmental Protection (ICPA Bucharest, Romania), using Romanian standard agrochemical methods, including SR 7184-13:2001 for soil pH [39], STAS 7184/21-82 for humus determination [51], nitrate nitrogen (N–NO_3_^−^) by internal validated procedure according to ICPA Methodology (1981, vol. 1, chapter 10, PT 46) [52], STAS 7184/19-82 for ammonium lactate extractable phosphorus (P_AL_) [53], and STAS 7184/18-80 [54], for ammonium lactate extractable potassium (K_AL_).

### 4.2. Experimental Design, Cultivars, and Fertiliser Treatments

Two raspberry cultivars (*Opal* and *Delniwa*) were monitored in each agroecosystem. In 2024, sampling was conducted under baseline management (no experimental fertiliser inputs). In 2025, the pre-established management zones were used to implement a fertilisation trial consisting of an unfertilised control and two bio-based fertiliser treatments (fertiliser 1 (FER1) and fertiliser 2 (FER2)). The two-year design reflected the sequential implementation of the research activities. The 2024 growing season was used to establish baseline isotope signatures under existing management conditions, whereas the fertilisation experiment was initiated in 2025 following the development, production, and characterisation of the experimental bio-based fertilisers (FER1 and FER2). This approach enabled the evaluation of fertiliser-related isotope responses relative to baseline conditions established during the previous season.

FER1 is a project-developed organic biofertiliser obtained via anaerobic digestion and applied in this study as the digestate fraction only. The material was derived from agricultural biomass produced on degraded, unproductive soils in the Valea Jiului coal mining area, Romania, where sorghum, soybean, and maize were cultivated as part of a phytoremediation-based land restoration strategy [55]. The resulting stems and leaves were subsequently subjected to experimental anaerobic digestion in order to obtain a biofertiliser designed to contribute to heavy metal stabilisation, moderation of soil acidity, and enhancement of carbon storage potential. The digestate provides plant-available nutrients and labile organic matter intended to support nutrition and stimulate soil biological activity. The material was designed to support nutrient cycling, microbial activity, and soil biological functioning while simultaneously contributing to soil improvement in degraded environments. Representative batches were chemically characterised for major nutrients and selected physicochemical parameters prior to application.

FER2 is a project-developed organo-mineral biocomposite formulated from a combination of mineral and industrial constituents, including alkaline and Ca–Mg–silicate-rich materials, together with organic fractions recovered from agro-industrial residues such as pomace- and yeast-derived by-products. The formulation was optimised using a statistical mixture-design approach, whereby different proportions of the constituent materials were evaluated to identify combinations providing a balanced performance in terms of pH buffering capacity, nutrient delivery, and functional organic properties. Conceived as a valorisation route for diverse agro-industrial waste streams, this material was reintegrated into the soil as an amendment aimed at improving key soil characteristics while supporting more sustainable agricultural practices. Its mode of action was designed to operate through both (i) chemical enhancement of soil conditions, via alkalinity and mineral nutrient inputs, and (ii) biological stimulation, through the addition of organic matter and bioactive or prebiotic components. From an agronomic perspective, FER2 can be broadly described as a solid organo-mineral amendment combining alkaline mineral constituents with organic matter recovered from agro-industrial by-products. The material was intended to provide both nutrient inputs and soil-conditioning effects through its mineral and organic fractions.

The fertilisation framework was informed by baseline agrochemical diagnostics of the 0–30 cm layer, indicating slightly alkaline soils at both sites, humus <4% (lower at Vlădești than INMA), lower available P at Vlădești, and higher K at INMA. Site-specific annual nutrient targets (expressed as N–P_2_O_5_–K_2_O) were used as a reference (85–50–65 kg ha^−1^ for Vlădești; 100–25–55 kg ha^−1^ for INMA). Application doses were calculated separately for FER1 and FER2 based on their respective nutrient composition, aiming to align with these targets as far as feasible. In 2025, fertilisers were applied in two split doses: once prior to vegetative growth and once before flowering.

**Figure 6:**
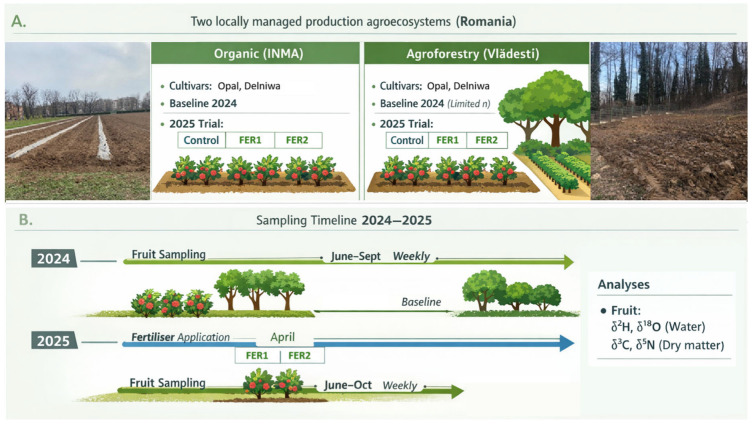
Study design and sampling overview. (**A**) Location of the two locally managed production agroecosystems in Romania: an organic raspberry plot (INMA, Bucharest) and an agroforestry-based plot (Vlădești, Vâlcea County)—conceptual schematics of the two production agroecosystems and the two monitored cultivars (*Opal* and *Delniwa*). (**B**) Sampling timeline across two seasons (2024–2025). In 2024, fruit sampling was conducted under baseline management (no experimental fertiliser application). In 2025, a fertiliser trial included unfertilised controls and plots treated with two bio-based fertilisers (FER1 and FER2). Ripe fruits were collected weekly during the harvest period for multi-isotope analysis (δ^2^H, δ^18^O, δ^13^C, δ^15^N).

The study design and sampling framework across years, cultivars, and treatments are illustrated in Figure 6.

### 4.3. Raspberry Fruit Sampling and Cultivar-Specific Harvest Dynamics

Raspberry fruits were collected by manual, sequential harvesting throughout the harvest season at both sites. Harvesting of both cultivars extended from June to October, with fruit ripening occurring progressively over ~20–30 days or longer; therefore, sampling was conducted in repeated passes during the season (typically every few days), preferentially in the morning or evening. Immediately after harvest, fruits were kept under cooled conditions for transport and transferred to the laboratory the same day. Due to the high perishability of raspberries, samples were frozen and stored at −18 °C prior to preparation. For isotopic analysis, berries were pooled into composite samples for each cultivar × agroecosystem × management zone × sampling date, with each composite integrating fruits from multiple plants within the same zone to reduce within-plant variability and obtain a representative plot-level signal.

**Figure 7:**
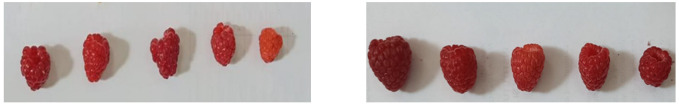
June morphometric measurements of raspberry fruits and plants: *Opal* (**left**) and *Delniwa* (**right**) berries (receptacle removed).

Cultivar-dependent differences in fruit size and seasonal dynamics were documented to contextualise sampling. *Delniwa* generally produced larger berries early in the season (June–July; 16.8–26.5 mm height, 16.5–23.3 mm diameter, and 3.3–5.6 g) compared with *Opal* (13.6–25.9 mm, 16.1–20.2 mm, and 2.1–5.3 g). Both cultivars exhibited reduced berry size and mass toward late season (August–October), consistent with end-of-harvest physiological conditions; *Delniwa* showed a more gradual decline, whereas *Opal* tended to decrease more sharply after July (Figure 7).

For each cultivar × zone × sampling date, composites were formed by pooling berries collected from multiple plants distributed along the zone to minimise plant-level variability. Soil and fruit sampling events were time-matched as closely as feasible to the phenological stage and management schedule.

### 4.4. Hydroclimatic Context and Water Availability During the 2024–2025 Seasons

Both experimental sites are located in southern Romania within a temperate-continental climatic setting, but they differ in landscape position and local microclimate. The INMA (Bucharest) organic plot is situated in a lowland terrace environment, where regional conditions are characterised by hot summers and cold winters. Historical meteorological records for the Bucharest area indicate a wide thermal amplitude (down to around −30 °C during severe winters and up to approximately +42 °C during extreme summer heat events), while recent multi-year averages report mean annual temperatures around 13 °C and annual precipitation on the order of 620–630 mm (2019–2022). The site lies on Quaternary loess/loessoid deposits typical of the southern plains, with soils commonly near-neutral to slightly alkaline and moderate organic matter at a regional scale [56]. In agreement with this setting, baseline agrochemical diagnostics of the 0–30 cm layer at INMA showed slightly alkaline topsoil (pH 7.23), humus content of 2.11%, available phosphorus (P_AL_ = 51 mg kg^−1^), and available potassium (K_AL_ = 175 mg kg^−1^) (Table 2).

The Vlădești agroforestry plot (Vâlcea County) is located at a higher elevation (322 m a.s.l.) adjacent to a forest edge characterised by a multi-strata canopy comprising a tall overstory and dense understory vegetation. This setting creates heterogeneous shading conditions across the experimental area and distinguishes the site from the more open-field conditions at INMA Bucharest. Baseline soil diagnostics (0–30 cm) indicated slightly alkaline conditions (pH 7.32), lower humus content (1.81%), lower available phosphorus (P_AL_ = 12 mg kg^−1^), and lower available potassium (K_AL_ = 84 mg kg^−1^) than at the INMA site (Table 2).

Seasonal hydroclimatic conditions differed between years and sites during the key phenological window (spring onset to late summer/early autumn). In 2024, April was mild and moderately wet at both locations (12.1–14 °C; 51–75 mm), followed by a relatively drier May at INMA (21–30 mm) than at Vlădești (42–50 mm). June rainfall was higher at INMA (76–100 mm) than at Vlădești (45–56 mm), while July was hot at both sites (≥24 °C) with moderate precipitation (51–75 mm). A pronounced late-summer drought occurred at Vlădești in August (<10 mm) under warm conditions (24–26 °C), whereas INMA received modest rainfall (21–30 mm) with very hot temperatures (>26 °C); September then shifted to a wet regime at both sites (Vlădești 101–125 mm; INMA 76–100 mm). In 2025, May was notably wet at both locations (Vlădești 101–125 mm; INMA 151–175 mm), June was very dry at Vlădești (6–20 mm) but wetter at INMA (51–75 mm), and July contrasted sharply (Vlădești 101–125 mm vs. INMA 21–40 mm). August 2025 was wet at Vlădești (76–100 mm) but exceptionally dry at INMA (<5 mm), with both sites receiving modest precipitation in September (21–30 mm). These differences provide essential context for interpreting seasonal variability in fruit-water δ^18^O and δ^2^H, which typically become more enriched under warm/dry conditions with higher evaporative demand and shift toward more depleted signatures following sustained rainfall and reduced evaporative enrichment.

Monthly temperature and precipitation summaries were compiled from the Romanian National Meteorological Administration (ANM) climatological characterisation portal (MeteoRomania), using the monthly and annual climatological summaries (https://www.meteoromania.ro/ accessed on 19 February 2026).

### 4.5. Sampling and Preparation of Soil Plant Samples for δ^13^C and δ^15^N Measurements

Raspberry fruits collected within each sampling campaign were pooled and homogenised by juice extraction using a centrifugal juicer (Moulinex, Jinan, China). From the homogenised juice, an aliquot of approximately 10 g was subsampled for each analytical unit intended for δ^13^C and δ^15^N determination. Subsamples were immediately frozen at −80 °C and subsequently freeze-dried for 72 h using a laboratory lyophilisation system (FreeZone 2.5 Plus, Labconco, Kansas City, MO, USA). The lyophilised material was then finely ground to a uniform powder, transferred to polypropylene microtubes (Kartell Sp.A, Noviglio, Italy), and kept dry until analysis by EA-IRMS (Flash EA 1112 HT, Thermo Scientific, Bremen, Germany).

### 4.6. Determination of δ^2^H and δ^18^O in Fruit Water by CF-IRMS

Hydrogen and oxygen isotope ratios were measured using a continuous-flow isotope ratio mass spectrometer (CF-IRMS; Delta V Plus, Thermo Scientific Corporation, Bremen, Germany) coupled to an isotope equilibration module (GasBench II; Thermo Scientific Corporation, Bremen, Germany) and processed using Isodat 3.0 software. For δ^18^O, a 500 µL aliquot of fruit water was equilibrated for 20 h at 24 °C with a gas mixture of 0.36% CO_2_ in He. The ^18^O/^16^O ratio was derived from ion currents at *m*/*z* 46 (^12^C^16^O^18^O) and *m*/*z* 44 (^12^C^16^O^16^O) of CO_2_ equilibrated with the sample water.

For δ^2^H, a 200 µL water aliquot was equilibrated via isotope exchange with H_2_ (2% H_2_ in He) in the presence of a Pt catalyst for at least 1 h at 25.0 ± 0.2 °C. Hydrogen isotope ratios were derived from *m*/*z* 3 (^2^H^1^H) and *m*/*z* 2 (^1^H^1^H) ion currents of H_2_ after equilibration.

Results are reported in δ notation (‰) relative to VSMOW:

$$\delta_{sample}\left(‰\right)=\left(\frac{R_{sample}}{R_{standard}}-1\right)\times 1000$$

At each analytical sequence, samples were bracketed with working laboratory water standards: B2192 (Zero Natural Water), IA-R064 (ISW Medium Natural Water), and OH32 (from participation in the Water Interlaboratory Comparison, WICO 2024). Working standards were periodically calibrated against primary reference materials VSMOW2, SLAP2, and GRESP (IAEA). Duplicate measurement reproducibility was better than ±0.2‰ for δ^18^O and ±1‰ for δ^2^H.

Fruit water extraction: water for δ^2^H and δ^18^O determination was obtained from fruit material using a freeze/lyophilisation approach with liquid nitrogen; from ~1.6 g of raspberry material, at least 1.5 mL of water was recovered.

### 4.7. Determination of δ^13^C and δ^15^N in Fruit Samples by EA-IRMS

Prepared fruit powders were weighed into tin capsules using an ultra-microbalance (Mettler Toledo XP6, Langacher, Switzerland). δ^13^C and δ^15^N were measured using an elemental analyser (Flash EA 1112 HT) coupled to a CF-IRMS (Delta V Plus, Thermo Scientific) via a ConFlo III interface. Samples were introduced using a MAS200 solids autosample (Thermo Scientific Corporation, Bremen, Germany). Combustion (Dumas) to CO_2_ and N_2_ was performed at 980 °C and 960 °C, respectively, with He 5.0 (99.999%) as the carrier gas. Reference and sample gases were diluted with ultra-pure helium before IRMS detection.

Isotope ratios are reported in δ notation (‰) relative to VPDB for carbon and AIR for nitrogen. Each run included certified reference materials (Iso-Analytical, Crewe, UK): (i) for Carbon: IA-R001 wheat flour (δ^13^C = −26.46 ± 0.26‰), IA-R004 corn flour (δ^13^C = −10.99 ± 0.06‰), IA-R041 alanine (δ^13^C = −23.33 ± 0.10‰); and (ii) for Nitrogen: IA-R001 wheat flour (δ^15^N = +2.55 ± 0.20‰), IA-R041 alanine (δ^15^N = −5.56 ± 0.14‰), IA-R045 ammonium sulphate (δ^15^N = −4.71 ± 0.07‰)

Analytical accuracy was ±0.40‰ for δ^15^N and ±0.40‰ for δ^13^C. Data processing was performed in Isodat 3.0.

### 4.8. Statistical Analysis

Descriptive statistics were calculated for all isotope variables (δ^15^N, δ^13^C, δ^18^O and δ^2^H), including means, standard deviations, and ranges for each production agroecosystem, cultivar, sampling year, and treatment category. Differences between production agroecosystems, growing seasons, and fertilisation categories were evaluated using the non-parametric Mann–Whitney U test, which does not require the assumption of normality and is suitable for comparisons between independent groups with unequal sample sizes. Comparisons were performed separately for each isotope variable (δ^15^N, δ^13^C, δ^18^O and δ^2^H), and statistical significance was accepted at *p* < 0.05. Results of the statistical comparisons are provided in Table S3 of the Supplementary Material.

Relationships among isotope variables were assessed using Pearson correlation coefficients calculated separately for each production agroecosystem based on the 2025 dataset.

Multivariate patterns in fruit isotope composition were explored using principal component analysis (PCA) based on the four isotope variables (δ^15^N, δ^13^C, δ^18^O and δ^2^H). PCA was used as an unsupervised exploratory technique to identify the major sources of variation within the isotope dataset and to evaluate the relative contribution of individual isotope variables. Prior to analysis, isotope variables were standardised within XLSTAT using the software’s mean-centring and unit variance scaling procedure, whereby the mean of each variable was subtracted from each observation, and the centred values were divided by the corresponding standard deviation. This standardisation was applied because the four isotope variables exhibited different numerical ranges and variances, and standardisation prevents variables with larger variances from disproportionately influencing the multivariate analyses. Consequently, all isotope variables contributed equally to the calculation of the principal components. PCA was performed using the correlation matrix implemented in XLSTAT. The number of retained principal components was evaluated based on the proportion of explained variance and inspection of the scree plot. The first two principal components, accounting for 78.1% of the total variance, were retained for interpretation and visualisation.

To evaluate the ability of the combined isotope fingerprint to differentiate predefined sample groups, discriminant analysis (DA) was subsequently performed using the same standardised isotope variables. Unlike PCA, which is an unsupervised method, DA is a supervised classification technique that maximises separation among predefined classes. In the present study, DA was used to assess discrimination among production agroecosystems and to evaluate the contribution of the combined isotope dataset to sample classification. Sample scores were visualised in canonical discriminant space to assess separation among production agroecosystems, growing seasons, and treatment categories.

All statistical analyses were performed using Addinsoft XLSTAT software version 2014.5.03 (Addinsoft Inc., New York, NY, USA).

## 5. Conclusions

This study demonstrates that raspberry fruits retain a distinct multi-isotope fingerprint that reflects differences between locally managed production agroecosystems. Across two growing seasons and two cultivars, production system effects represented the dominant source of variation in fruit isotope composition.

Among the investigated isotope tracers, δ^15^N provided the strongest discrimination between agroecosystems and was consistent with differences in nitrogen cycling and nutrient dynamics between the two production systems. However, because direct isotopic measurements of soil nitrogen pools and potential nitrogen sources were not available, the observed δ^15^N differences should be interpreted as reflecting the combined influence of nitrogen sources, soil nitrogen transformations, and plant uptake processes rather than a single identifiable mechanism. Fruit water isotopes (δ^2^H and δ^18^O) were consistent with hydroclimatic variability and evaporative enrichment processes occurring along the soil–plant–atmosphere continuum, as supported by the comparison between the Raspberry Fruit Water Line and the local meteoric water lines. Carbon isotope composition (δ^13^C) provided complementary information that may reflect differences in plant physiological responses, water-use conditions, and microenvironmental variability.

Under the conditions investigated here, short-term fertiliser applications produced only modest isotopic shifts relative to the broader agroecosystem signal and the variability associated with seasonal hydroclimatic conditions. Multivariate analyses indicated that the combined four-isotope dataset effectively differentiated the investigated organic and agroforestry production systems, although the observed discrimination was driven primarily by δ^15^N, while the remaining isotope variables contributed complementary information.

An important limitation of the present work is the absence of direct isotopic characterisation of soil nitrogen pools, fertiliser materials and other potential nitrogen sources. Consequently, the mechanisms responsible for the observed δ^15^N differences cannot be resolved unequivocally. Future studies integrating fruit isotope fingerprints with soil biogeochemical measurements, source isotope characterisation and nitrogen cycling indicators would provide a more comprehensive understanding of the processes underlying agroecosystem-specific isotope signatures.

Overall, these findings highlight the value of multi-isotope approaches as integrative tools for relating fruit composition to environmental conditions, plant physiological responses, and agroecosystem processes. Beyond their relevance for food authentication and traceability, isotope fingerprints may provide useful indicators of agroecosystem functioning and resilience. In a broader food-systems context, multi-isotope approaches may contribute to more transparent horticultural supply chains by supporting traceability, sustainability claims, and the independent verification of production-system characteristics.

## Figures and Tables

**Table 1:** Stable isotope ratios (mean ± SD) of raspberry fruit by production agroecosystem, year, cultivar, and treatment category.

Production Agroecosystem	Year	Cultivar	Treatment	δ^2^H (‰)	δ^18^O (‰)	δ^13^C (‰)	δ^15^N (‰)
Agroforestry	2024	*Delniwa*	Control (*n* = 5)	−13.77 ± 1.53	3.03 ± 0.29	−24.79 ± 0.17)	−0.93 ± 0.21
		*Opal*	Control (*n* = 5)	−10.11 ± 3.10	2.92 ± 0.31	−24.84 ± 0.13	−1.33 ± 0.27
	2025	*Delniwa*	All (*n* = 16)	−18.29 ± 5.94	3.29 ± 2.02	−25.71 ± 0.69	−1.17 ± 0.44
			Control (*n* = 10)	−18.66 ± 6.02	3.27 ± 1.84	−25.82 ± 0.69	−1.04 ± 0.36
			Fertilised (*n* = 6)	−17.67 ± 6.31	3.33 ± 2.47	−25.51 ± 0.71	−1.39 ± 0.50
		*Opal*	All (*n* = 17)	−20.90 ± 8.09	1.67 ± 2.18	−26.30 ± 0.73	−1.00 ± 1.18
			Control (*n* = 14)	−20.23 ± 8.24	1.70 ± 2.18	−26.19 ± 0.61	−1.17 ± 1.20
			Fertilised (*n* = 3)	−24.05 ± 7.99	1.53 ± 2.69	−26.80 ± 1.17	−0.16 ± 0.67
Organic	2024	*Delniwa*	Control (*n* = 13)	−22.96 ± 10.29	3.11 ± 3.15	−24.93 ± 1.20	7.62 ± 1.04
	2024	*Opal*	Control (*n* = 13)	−24.24 ± 8.15	3.08 ± 3.28	−24.44 ± 1.36	7.96 ± 0.98
	2025	*Delniwa*	All (*n* = 30)	−24.32 ± 11.05	4.34 ± 3.26	−25.32 ± 0.80	4.58 ± 0.99
			Control (*n* = 12)	−24.38 ± 12.56	4.35 ± 3.67	−25.33 ± 0.81	4.80 ± 1.09
			Fertilised (*n* = 18)	−24.28 ± 10.31	4.33 ± 3.06	−25.31 ± 0.82	4.43 ± 0.92
		*Opal*	All (*n* = 31)	−24.40 ± 9.65	4.34 ± 3.01	−25.29 ± 1.05	5.03 ± 1.37
			Control (*n* = 13)	−24.38 ± 12.42	4.18 ± 3.78	−25.33 ± 1.03	5.32 ± 1.66
			Fertilised (*n* = 18)	−24.41 ± 7.45	4.45 ± 2.42	−25.27 ± 1.09	4.82 ± 1.12

Values are mean ± SD (‰). “All” pools Control and Fertilised samples; “Fertilised” pools FER1 and FER2 treatments. Individual sample values are provided in Table S1 (Supplementary Material). In 2024, fertiliser treatments were not applied; therefore, only Control values are reported.

**Table 2:** Key site descriptors of the locally managed production agroecosystems.

Descriptor	Organic	Agroforestry
Location	INMA, Bucharest, Romania	Vlădești, Vâlcea County, Romania
Coordinates (WGS84)	44°30′01″ N, 26°04′19″ E	45°07′11″ N, 24°17′33″ E
Altitude (m a.s.l.)	60	322
Soil pH (0–30 cm)	7.23	7.32
Humus (%)	2.11	1.81
Available phosphorus, P_AL_ (mg kg^−1^)	51	12
Available potassium, K_AL_ (mg kg^−1^)	175	84
Nitrate nitrogen (NO_3_^−^–N)	Very high	Very high
Irrigation regime	Rainfed (no irrigation applied during the study)	Rainfed (no irrigation applied during the study)
Production system	Organic management	Agroforestry-based raspberry production
Canopy structure	Open-field cultivation	Forest-edge, multi-strata canopy providing heterogeneous shading conditions

Source: Coordinates and altitude were obtained from Google Earth Pro. Soil pH, humus content, available phosphorus (P_AL_, ammonium lactate extractable phosphorus), available potassium (K_AL_, ammonium lactate extractable potassium), and nitrate nitrogen (NO_3_^−^–N) were determined from agrochemical analyses of composite soil samples collected from the 0–30 cm layer prior to fertiliser application. Management descriptors (irrigation regime, production system, and canopy structure) were based on field observations and experimental records.

## Data Availability

The original contributions presented in this study are included in the article/Supplementary Materials. Further inquiries can be directed to the corresponding author.

## Supplementary Materials

The following supporting information can be downloaded at: https://www.mdpi.com/article/10.3390/molecules31142459/s1, Figure S1: Local meteoric water lines (LMWLs) derived from precipitation isotope monitoring in the Vlădești/Vâlcea region for 2024 and 2025. Differences in slope and intercept between years reflect interannual variability in hydroclimatic conditions and moisture-source characteristics at the regional scale; Figure S2: Seasonal variation in precipitation δ^2^H and δ^18^O values in the Vlădești/Vâlcea region during 2024, shown together with monthly precipitation amount and mean air temperature. The data illustrate the coupling between hydroclimatic conditions and meteoric water isotope composition over the growing season; Figure S3: Dual-isotope plot (δ^2^H vs. δ^18^O) showing raspberry fruit water isotopes from agroforestry ecosystems plotted against the global meteoric water line (GMWL) and local meteoric water lines for 2024 and 2025 in the Valdesti area; Figure S4: d-excess values (d = δ^2^H − 8·δ^18^O) for precipitation in the Vlădești/Vâlcea region and raspberry fruit water. Filled symbols correspond to 2024 samples and open symbols to 2025 samples. The dashed horizontal line marks d-excess = 10‰ (intercept of the Global Meteoric Water Line). Boxes show medians and interquartile ranges; points represent individual samples; Table S1: Stable isotope ratios of raspberry fruit samples by production agroecosystem, year and cultivar; Table S2: Pearson correlation coefficients (r) among carbon, nitrogen and water isotope ratios in raspberry fruit collected in 2025. Correlations are shown separately for organic and agroforestry production agroecosystems. Table S3: Results of Mann–Whitney U tests for isotope variables (δ^15^N, δ^13^C, δ^18^O and δ^2^H).
