# Supplementary material for: A Multi-Isotope Approach (δ2H, δ18O, δ13C, δ15N) for Discriminating Raspberry Production Systems and Assessing Agroecosystem Functioning
Source: Molecules. 2026 Jul 14;31(14):2459. doi: 10.3390/molecules31142459 (PMC13414084; doi:10.3390/molecules31142459)
Supplement: Supplementary file 1 [file molecules-31-02459-s001.zip › molecules-4330775-supplementary.pdf]

SUPPLEMENTARY MATERIAL

A Multi-Isotope Approach (δ^2H, $\delta^{18}O$, $\delta^{13}C$, $\delta^{15}N$) for Discriminating Raspberry Production Systems and Assessing Agroecosystem Functioning

Roxana Elena Ionete [1], Diana Costinel [1], Ana Maria Simionescu [1], Marius Gheorghe Miricioiu [1], Augustina Pruteanu [2], Aura Irina Istrate [3] and Oana Romina Botoran [1,*]

[1] National Research and Development Institute for Cryogenic and Isotopic Technologies—ICSI Râmnicu Vâlcea, 4th Uzinei Street, P.O. Box Raureni 7, 240050 Râmnicu Vâlcea, Romania; roxana.ionete@icsi.ro (R.E.I.); diana.costinel@icsi.ro (D.C.); ana.simionescu@icsi.ro (A.M.S.); marius.miricioiu@icsi.ro (M.G.M.)

[2] National Institute of Research—Development for Machines and Installations Designed for Agriculture and Food Industry—INMA, 013813 Bucharest, Romania; pruteanu@inma.ro

[3] Faculty of Biotechnical Systems Engineering, National University of Science and Technology Politehnica Bucharest, Splaiul Independentei 313, 060042 Bucharest, Romania; irina_aura.istrate@upb.ro

* Correspondence: oana.dinca@icsi.ro

Data overview

Raspberry fruits were sampled across two locally managed production agroecosystems—an organic system at INMA (Bucharest; minimally irrigated) and a rainfed agroforestry system at Vlădești (Vâlcea)—over two consecutive growing seasons (2024–2025). Sampling encompassed two cultivars (*Opal* and *Delniwa*) and covered the main harvest period from early summer to early autumn. In 2025, both unfertilised control plots and plots receiving bio-based fertilisers (FER1 and FER2) were included.

The isotope dataset comprised measurements of δ^2H and $\delta^{18}O$ in fruit water and $\delta^{13}C$ and $\delta^{15}N$ in bulk dried fruit material. The dataset consisted of 130 fruit samples, including 87 samples from the organic agroecosystem and 43 samples from the agroforestry agroecosystem.

Individual isotope values for all samples are reported in Table S1.

Table S1. Stable isotope ratios of raspberry fruit samples by production agroecosystem, year and cultivar.

Cultivation system	Raspberry variety	Treatment	Areal	Sampling data	$\delta^{18}O$ (‰)	δ^2H (‰)	$\delta^{13}C$ (‰)	$\delta^{15}N$ (‰)
organic	OPAL	Natural	INMA Bucuresti	6/12/2024	2.79	−17.48	−25.02	7.60
organic	OPAL	Natural	INMA Bucuresti	6/18/2024	2.31	−17.51	−26.17	7.62
organic	OPAL	Natural	INMA Bucuresti	6/26/2024	5.06	−21.31	−26.15	7.69
organic	OPAL	Natural	INMA Bucuresti	7/5/2024	3.75	−26.83	−25.77	7.14
organic	OPAL	Natural	INMA Bucuresti	7/11/2024	5.06	−26.55	−25.66	7.30
organic	OPAL	Natural	INMA Bucuresti	7/24/2024	2.31	−19.27	−23.86	6.09
organic	OPAL	Natural	INMA Bucuresti	7/29/2024	3.70	−21.49	−24.09	7.42
organic	OPAL	Natural	INMA Bucuresti	8/5/2024	7.30	−14.43	−22.82	9.62
organic	OPAL	Natural	INMA Bucuresti	8/13/2024	5.48	−20.87	−22.62	9.16

Cultivation system	Raspberry variety	Treatment	Areal	Sampling data	δ18O (‰)	δ2H (‰)	δ13C (‰)	δ15N (‰)
organic	OPAL	Natural	INMA Bucuresti	8/30/2024	7.80	−18.72	−22.82	9.38
organic	OPAL	Natural	INMA Bucuresti	9/1/2024	−1.38	−32.79	−22.93	7.92
organic	OPAL	Natural	INMA Bucuresti	10/1/2024	−1.84	−36.63	−25.61	8.31
organic	OPAL	Natural	INMA Bucuresti	11/7/2024	−2.30	−41.21	−24.18	8.18
organic	DELNIWA	Natural	INMA Bucuresti	6/12/2024	2.41	−20.31	−24.22	7.59
organic	DELNIWA	Natural	INMA Bucuresti	6/18/2024	2.72	−14.06	−26.46	7.27
organic	DELNIWA	Natural	INMA Bucuresti	6/26/2024	6.38	−10.76	−25.69	7.29
organic	DELNIWA	Natural	INMA Bucuresti	7/2/2024	3.95	−19.54	−26.38	7.67
organic	DELNIWA	Natural	INMA Bucuresti	7/5/2024	0.57	−32.63	−25.08	7.89
organic	DELNIWA	Natural	INMA Bucuresti	7/11/2024	4.94	−28.02	−25.80	8.09
organic	DELNIWA	Natural	INMA Bucuresti	7/24/2024	2.53	−17.60	−24.06	6.71
organic	DELNIWA	Natural	INMA Bucuresti	7/29/2024	3.66	−17.97	−24.76	6.62
organic	DELNIWA	Natural	INMA Bucuresti	8/5/2024	8.39	−11.59	−23.1	9.77
organic	DELNIWA	Natural	INMA Bucuresti	8/30/2024	7.58	−16.06	−22.92	9.59
organic	DELNIWA	Natural	INMA Bucuresti	9/1/2024	−0.89	−35.18	−23.97	7.36
organic	DELNIWA	Natural	INMA Bucuresti	10/1/2024	−0.90	−30.11	−25.64	6.41
organic	DELNIWA	Natural	INMA Bucuresti	11/7/2024	−0.93	−44.71	−26.04	6.8
organic	OPAL	Natural	INMA Bucuresti	6/11/2025	0.92	−24.03	−26.88	7.59
organic	OPAL	Natural	INMA Bucuresti	6/26/2025	7.01	−24.30	−25.56	7.23
organic	OPAL	Natural	INMA Bucuresti	7/2/2025	7.30	−8.35	−25.69	5.65
organic	OPAL	Natural	INMA Bucuresti	7/7/2025	5.89	−18.85	−25.61	4.95
organic	OPAL	Natural	INMA Bucuresti	7/10/2025	5.71	−15.70	−26.03	7.83
organic	OPAL	Natural	INMA Bucuresti	7/15/2025	3.77	−25.67	−26.5	3.67
organic	OPAL	Natural	INMA Bucuresti	7/21/2025	2.63	−29.28	−26.14	3.91
organic	OPAL	Natural	INMA Bucuresti	7/28/2025	7.33	−17.25	−24.65	6.43
organic	OPAL	Natural	INMA Bucuresti	8/6/2025	6.00	−24.25	−24.85	4.69
organic	OPAL	Natural	INMA Bucuresti	8/13/2025	7.66	−15.23	−24.74	4.47

Cultivation system	Raspberry variety	Treatment	Areal	Sampling data	δ¹⁸O (‰)	δ²H (‰)	δ¹³C (‰)	δ¹⁵N (‰)
organic	OPAL	Natural	INMA Bucuresti	8/18/2025	3.00	−24.19	−23.62	3.66
organic	OPAL	Natural	INMA Bucuresti	9/1/2025	3.30	−29.41	−23.47	2.73
organic	OPAL	Natural	INMA Bucuresti	10/1/2025	−6.19	−60.46	−25.54	6.32
organic	Delniwa	Natural	INMA Bucuresti	6/26/2025	8.03	−13.6	−25.74	5.43
organic	Delniwa	Natural	INMA Bucuresti	7/2/2025	6.71	−10.42	−24.26	6.61
organic	Delniwa	Natural	INMA Bucuresti	7/7/2025	5.05	−24.31	−26.31	4.51
organic	Delniwa	Natural	INMA Bucuresti	7/10/2025	4.79	−20.85	−26.03	6.99
organic	Delniwa	Natural	INMA Bucuresti	7/15/2025	4.48	−30.62	−25.71	3.51
organic	Delniwa	Natural	INMA Bucuresti	7/21/2025	3.53	−26.73	−25.33	4.72
organic	Delniwa	Natural	INMA Bucuresti	7/28/2025	7.52	−11.06	−25.43	3.94
organic	Delniwa	Natural	INMA Bucuresti	8/6/2025	4.43	−32.58	−25.38	3.96
organic	Delniwa	Natural	INMA Bucuresti	8/13/2025	7.01	−17.49	−25.12	4.66
organic	Delniwa	Natural	INMA Bucuresti	8/18/2025	3.55	−21.86	−24.13	3.68
organic	Delniwa	Natural	INMA Bucuresti	9/1/2025	3.12	−26.03	−24.03	4.54
organic	Delniwa	Natural	INMA Bucuresti	10/1/2025	−6.08	−57.03	−26.43	5.05
organic	OPAL	FER1	INMA Bucuresti	6/26/2025	7.71	−15.68	−26.18	6.19
organic	OPAL	FER1	INMA Bucuresti	7/2/2025	5.75	−15.21	−26.35	5.63
organic	OPAL	FER1	INMA Bucuresti	7/10/2025	5.20	−17.52	−26.22	5.72
organic	OPAL	FER1	INMA Bucuresti	7/15/2025	3.27	−32.55	−26.44	6.11
organic	OPAL	FER1	INMA Bucuresti	7/21/2025	1.58	−33.71	−26.49	4.24
organic	OPAL	FER1	INMA Bucuresti	7/28/2025	4.75	−21.17	−24.81	3.77
organic	OPAL	FER1	INMA Bucuresti	8/6/2025	2.86	−27.24	−24.82	3.94
organic	OPAL	FER1	INMA Bucuresti	8/13/2025	7.74	−21.74	−24.21	3.99
organic	OPAL	FER1	INMA Bucuresti	9/1/2025	2.47	−29.13	−23.06	3.71
organic	OPAL	FER2	INMA Bucuresti	6/26/2025	7.55	−15.4	−24.65	7.25
organic	OPAL	FER2	INMA Bucuresti	7/2/2025	5.05	−24.7	−24.85	5.62
organic	OPAL	FER2	INMA Bucuresti	7/10/2025	4.39	−23.63	−26.59	6.19

Cultivation system	Raspberry variety	Treatment	Areal	Sampling data	δ18O (‰)	δ2H (‰)	δ13C (‰)	δ15N (‰)
organic	OPAL	FER2	INMA Bucuresti	7/15/2025	2.00	−36.47	−25.59	3.99
organic	OPAL	FER2	INMA Bucuresti	7/21/2025	1.73	−32.33	−26.48	4.30
organic	OPAL	FER2	INMA Bucuresti	7/28/2025	4.95	−23.71	−25.41	3.71
organic	OPAL	FER2	INMA Bucuresti	8/6/2025	1.70	−28.94	−24.87	3.92
organic	OPAL	FER2	INMA Bucuresti	8/13/2025	9.25	−10.51	−24.30	4.03
organic	OPAL	FER2	INMA Bucuresti	9/1/2025	2.13	−29.80	−23.52	4.53
organic	Delniwa	FER1	INMA Bucuresti	6/26/2025	10.41	−5.36	−25.52	5.37
organic	Delniwa	FER1	INMA Bucuresti	7/2/2025	8.07	−7.88	−24.34	4.15
organic	Delniwa	FER1	INMA Bucuresti	7/10/2025	3.87	−24.97	−26.03	6.24
organic	Delniwa	FER1	INMA Bucuresti	7/15/2025	2.07	−35.88	−25.02	5.98
organic	Delniwa	FER1	INMA Bucuresti	7/21/2025	−0.18	−38.52	−24.91	4.02
organic	Delniwa	FER1	INMA Bucuresti	7/28/2025	4.37	−23.18	−25.59	3.67
organic	Delniwa	FER1	INMA Bucuresti	8/6/2025	−0.14	−33.69	−25.77	3.82
organic	Delniwa	FER1	INMA Bucuresti	8/13/2025	8.45	−13.38	−25.00	3.90
organic	Delniwa	FER1	INMA Bucuresti	9/1/2025	2.90	−28.48	−24.49	3.67
organic	Delniwa	FER2	INMA Bucuresti	6/26/2025	5.60	−14.99	−24.64	5.57
organic	Delniwa	FER2	INMA Bucuresti	7/2/2025	7.28	−14.20	−26.08	4.88
organic	Delniwa	FER2	INMA Bucuresti	7/10/2025	4.35	−22.76	−25.39	5.09
organic	Delniwa	FER2	INMA Bucuresti	7/15/2025	1.97	−37.61	−27.38	4.46
organic	Delniwa	FER2	INMA Bucuresti	7/21/2025	0.42	−36.69	−25.76	4.54
organic	Delniwa	FER2	INMA Bucuresti	7/28/2025	4.49	−23.42	−25.85	3.99
organic	Delniwa	FER2	INMA Bucuresti	8/6/2025	3.19	−27.52	−25.20	3.69
organic	Delniwa	FER2	INMA Bucuresti	8/13/2025	7.55	−17.80	−25.00	3.93
organic	Delniwa	FER2	INMA Bucuresti	9/1/2025	3.35	−30.65	−23.67	2.77
agroforestry	OPAL	Natural	Vladesti - Valcea	6/12/2024	2.70	−7.19	−24.92	−1.50
agroforestry	OPAL	Natural	Vladesti - Valcea	6/27/2024	2.52	−7.43	−24.78	−1.20
agroforestry	OPAL	Natural	Vladesti - Valcea	7/2/2024	2.98	−9.36	−25.01	−0.97

Cultivation system	Raspberry variety	Treatment	Areal	Sampling data	δ18O (‰)	δ2H (‰)	δ13C (‰)	δ15N (‰)
agroforestry	OPAL	Natural	Vladesti - Valcea	7/15/2024	3.30	−12.32	−24.66	−1.32
agroforestry	OPAL	Natural	Vladesti - Valcea	7/27/2024	3.12	−14.26	−24.84	−1.66
agroforestry	DELNIWA	Natural	Vladesti - Valcea	6/12/2024	2.99	−12.26	−24.74	−0.78
agroforestry	DELNIWA	Natural	Vladesti - Valcea	6/27/2024	2.74	−14.58	−24.93	−0.67
agroforestry	DELNIWA	Natural	Vladesti - Valcea	7/2/2024	3.03	−12.56	−24.61	−1.21
agroforestry	DELNIWA	Natural	Vladesti - Valcea	7/15/2024	3.50	−15.99	−24.68	−0.96
agroforestry	DELNIWA	Natural	Vladesti - Valcea	7/27/2024	2.88	−13.46	−25.00	−1.03
agroforestry	OPAL	Natural	Vladesti - Valcea	6/10/2025	−1.03	−20.60	−27.52	−2.37
agroforestry	OPAL	Natural	Vladesti - Valcea	6/14/2025	0.77	−18.90	−26.92	0.62
agroforestry	OPAL	Natural	Vladesti - Valcea	6/18/2025	1.70	−18.09	−26.93	0.32
agroforestry	OPAL	Natural	Vladesti - Valcea	6/22/2025	2.04	−19.00	−25.80	−1.34
agroforestry	OPAL	Natural	Vladesti - Valcea	6/23/2025	3.08	−18.46	−26.05	0.07
agroforestry	OPAL	Natural	Vladesti - Valcea	6/25/2025	4.44	−12.23	−26.59	−0.52
agroforestry	OPAL	Natural	Vladesti - Valcea	6/30/2025	4.33	−10.23	−25.33	−0.37
agroforestry	OPAL	Natural	Vladesti - Valcea	7/2/2025	4.09	−13.48	−25.85	−0.43
agroforestry	OPAL	Natural	Vladesti - Valcea	7/8/2025	4.46	−14.42	−25.70	−0.62
agroforestry	OPAL	Natural	Vladesti - Valcea	7/10/2025	1.31	−14.83	−25.86	−2.93
agroforestry	OPAL	Natural	Vladesti - Valcea	7/14/2025	1.71	−21.18	−26.18	−1.86
agroforestry	OPAL	Natural	Vladesti - Valcea	7/18/2025	−1.81	−37.98	−26.01	−3.01
agroforestry	OPAL	Natural	Vladesti - Valcea	7/21/2025	−0.47	−34.24	−25.60	−2.23
agroforestry	OPAL	Natural	Vladesti - Valcea	8/6/2025	−0.84	−29.54	−26.38	−1.77
agroforestry	Delniwa	Natural	Vladesti - Valcea	6/14/2025	1.20	−23.26	−26.66	−1.17
agroforestry	Delniwa	Natural	Vladesti - Valcea	6/18/2025	3.08	−18.64	−25.92	−0.75
agroforestry	Delniwa	Natural	Vladesti - Valcea	6/22/2025	2.01	−21.23	−26.83	−0.86
agroforestry	Delniwa	Natural	Vladesti - Valcea	6/23/2025	2.33	−24.28	−26.46	−1.22
agroforestry	Delniwa	Natural	Vladesti - Valcea	6/25/2025	4.85	−17.16	−25.36	−1.29
agroforestry	Delniwa	Natural	Vladesti - Valcea	6/30/2025	2.37	−23.06	−26.15	−1.12

Cultivation system	Raspberry variety	Treatment	Areal	Sampling data	δ18O (‰)	δ2H (‰)	δ13C (‰)	δ15N (‰)
agroforestry	Delniwa	Natural	Vladesti - Valcea	6/2/2025	4.76	−20.05	−24.89	−0.69
agroforestry	Delniwa	Natural	Vladesti - Valcea	6/8/2025	7.34	−4.62	−25.33	−1.81
agroforestry	Delniwa	Natural	Vladesti - Valcea	6/10/2025	2.38	−12.57	−25.61	−0.82
agroforestry	Delniwa	Natural	Vladesti - Valcea	6/6/2025	2.33	−21.71	−25.03	−0.67
agroforestry	OPAL	FER1	Vladesti - Valcea	7/8/2025	4.58	−15.57	−25.98	−0.25
agroforestry	OPAL	FER1	Vladesti - Valcea	7/10/2025	0.50	−25.14	−28.14	0.55
agroforestry	OPAL	FER1	Vladesti - Valcea	7/21/2025	−0.50	−31.44	−26.27	−0.79
agroforestry	Delniwa	FER2	Vladesti - Valcea	7/8/2025	6.22	−13.71	−26.7	−0.49
agroforestry	Delniwa	FER2	Vladesti - Valcea	7/10/2025	2.21	−20.66	−25.84	−1.58
agroforestry	Delniwa	FER2	Vladesti - Valcea	8/6/2025	0.38	−27.34	−25.50	−1.30
agroforestry	Delniwa	FER1	Vladesti - Valcea	7/8/2025	6.45	−9.05	−24.60	−1.42
agroforestry	Delniwa	FER1	Vladesti - Valcea	7/10/2025	1.83	−19.42	−25.12	−2.00
agroforestry	Delniwa	FER1	Vladesti - Valcea	8/6/2025	2.88	−15.86	−25.29	−1.54

Across the full dataset, isotopic variability was substantial, particularly for the water-related isotopes. Fruit water δ2H values ranged from −60.46 to −4.62‰, while δ18O values spanned −6.19 to 10.41‰, indicating strong hydroclimatic control and variable evaporative enrichment during water transfer along the soil–plant–atmosphere continuum. Carbon isotope ratios (δ13C) remained within the expected range for C_3 crops (−28.14 to −22.62‰) across production systems, years and cultivars, reflecting typical photosynthetic discrimination under varying environmental conditions.

Nitrogen isotope ratios exhibited the strongest contrast between production agroecosystems. Across all samples, fruit δ15N values ranged from −3.01 to 0.62‰ in the agroforestry agroecosystem and from 2.73 to 9.77‰ in the organic agroecosystem, with virtually no overlap between systems. This pronounced system-level separation is already evident in the group-level summaries (Table 2), where agroforestry fruit consistently shows depleted δ15N values close to atmospheric nitrogen signatures, whereas organic fruit displays substantially enriched values. This contrast provides the framework for the subsequent analyses of overall dataset patterns, baseline isotope signatures in unfertilised controls, and fertiliser-related isotopic shifts

Fertiliser effects across isotopes

Table S2. Pearson correlation coefficients (r) among carbon, nitrogen and water isotope ratios in raspberry fruit collected in 2025. Correlations are shown separately for organic and agroforestry production agroecosystems.

Production system	Isotope pair	r	p-value	n
Organic	δ13C *vs* δ18O	0.157	0.227	61
Organic	δ13C *vs* δ2H	0.152	0.244	61
Organic	δ15N *vs* δ13C	−0.363	0.004	61
Agroforestry	δ13C *vs* δ18O	0.436	0.011	33

Agroforestry	$\delta^{13}C$ *vs* $\delta^{2}H$	0.264	0.138	33
Agroforestry	$\delta^{15}N$ *vs* $\delta^{13}C$	−0.296	0.094	33

Correlations were computed using Pearson's r. All analyses were performed on 2025 samples only, pooling fertilised and unfertilised fruits within each production system.

Pearson correlation analysis revealed weak to moderate coupling among isotope tracers, with a positive relationship between $\delta^{13}C$ and $\delta^{18}O$ in the agroforestry system and generally weak correlations between nitrogen and carbon isotopes in both production systems, indicating that fertilisation did not fundamentally alter the structure of isotope relationships.

Statistical comparison of isotope compositions among production systems, years, and fertilisation categories

To support the interpretation of isotopic differences observed among production agroecosystems, growing seasons, and fertilisation categories, non-parametric Mann–Whitney U tests were performed for each isotope variable. Comparisons were conducted because isotope data were not always normally distributed and sample sizes were unbalanced among groups. Statistical significance was accepted at $p < 0.05$. Results are summarised in Table S3.

Table S3. Results of Mann–Whitney U tests for isotope variables ($\delta^{15}N$, $\delta^{13}C$, $\delta^{18}O$ and $\delta^{2}H$).

Comparison	$\delta^{15}N$	$\delta^{13}C$	$\delta^{18}O$	$\delta^{2}H$
Organic *versus* Agroforestry (all samples)	<0.001	0.004	0.001	<0.001
Organic 2024 *versus* Organic 2025	<0.001	0.008	0.094	0.848
Agroforestry 2024 *versus* Agroforestry 2025	0.752	<0.001	0.177	<0.001
Organic 2025 Control *versus* Fertilised	0.288	0.889	0.509	0.533

Linking fruit water to precipitation isotope baselines

To place fruit water isotope patterns in a meteoric context, precipitation isotope monitoring from the Vlădești/Vâlcea region was used as a regional baseline. Local meteoric water lines (LMWLs) were derived from precipitation isotope measurements for 2024 and 2025 (Figure S1). The resulting lines differed between years, with variations in slope and intercept reflecting interannual changes in hydroclimatic conditions and regional moisture-source characteristics. Seasonal variability in precipitation isotopes during 2024 further illustrates the coupling between hydroclimatic conditions and meteoric water composition (Figure S2). Periods of higher temperature and lower precipitation corresponded to more enriched meteoric water signatures, whereas wetter intervals were associated with more depleted isotopic values.

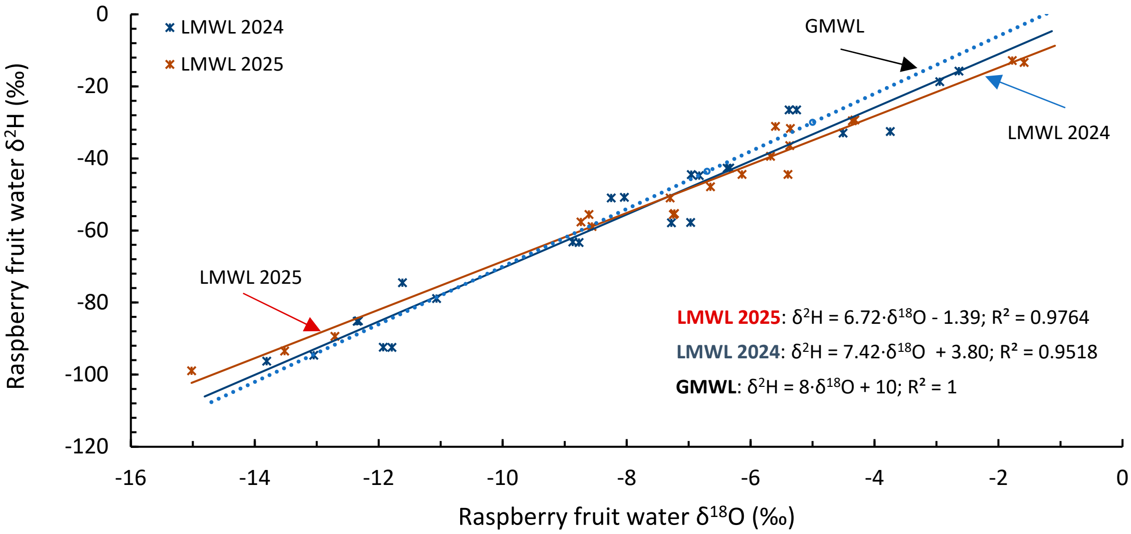

Figure S1. Local meteoric water lines (LMWLs) derived from precipitation isotope monitoring in the Vlădești/Vâlcea region for 2024 and 2025. Differences in slope and intercept between years reflect interannual variability in hydroclimatic conditions and moisture-source characteristics at the regional scale.

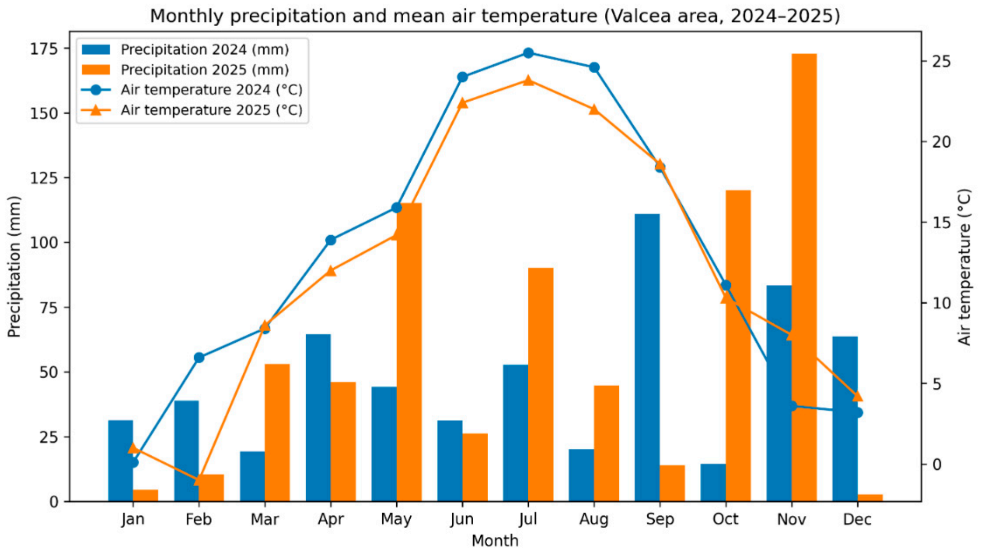

Figure S2. Seasonal variation in precipitation δ^2H and $\delta^{18}O$ values in the Vlădești/Vâlcea region during 2024, shown together with monthly precipitation amount and mean air temperature. The data illustrate the coupling between hydroclimatic conditions and meteoric water isotope composition over the growing season.

When fruit water isotopes were plotted together with the Vâlcea LMWLs (Figure S3), fruit values systematically fell below meteoric lines in dual-isotope space. This consistent deviation indicates that evaporative enrichment during soil evaporation, plant transpiration and fruit development dominates the isotopic composition of raspberry fruit water rather than direct inheritance of precipitation isotope signatures.

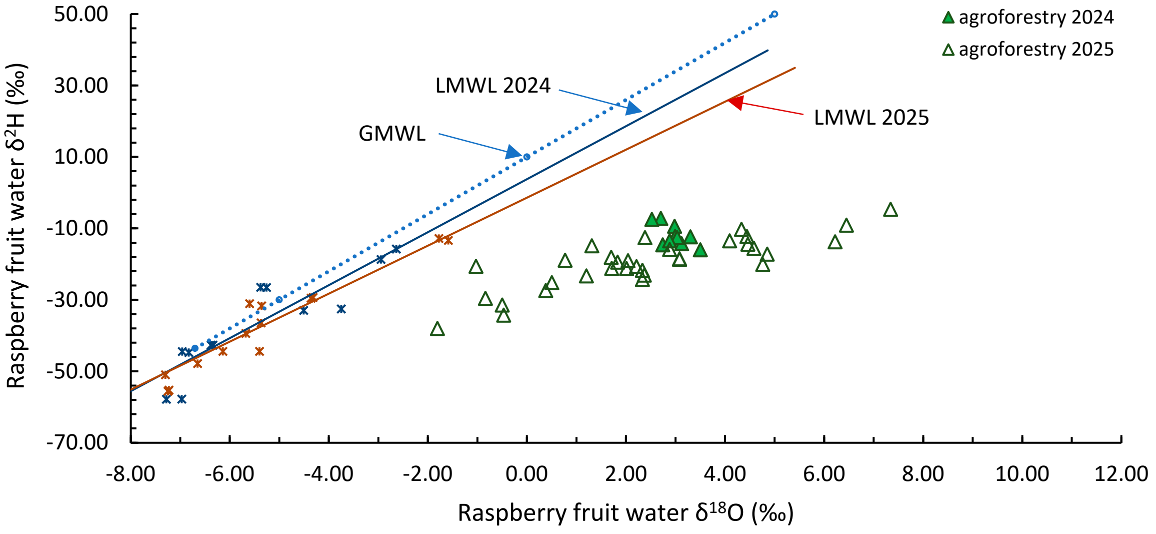

Figure S3. Dual-isotope plot (δ^2H *vs* $\delta^{18}O$) showing raspberry fruit water isotopes from agroforestry ecosystems plotted against the global meteoric water line (GMWL) and local meteoric water lines for 2024 and 2025 in the Valdesti area.

The contrast between meteoric water and fruit water is further highlighted by d-excess values (Figure S4). Precipitation generally exhibits d-excess values close to the Global Meteoric Water Line intercept (≈10‰), whereas fruit water displays markedly lower d-excess values. Such depletion is characteristic of waters that have undergone non-equilibrium evaporation within the soil–plant–atmosphere continuum prior to or during fruit development.

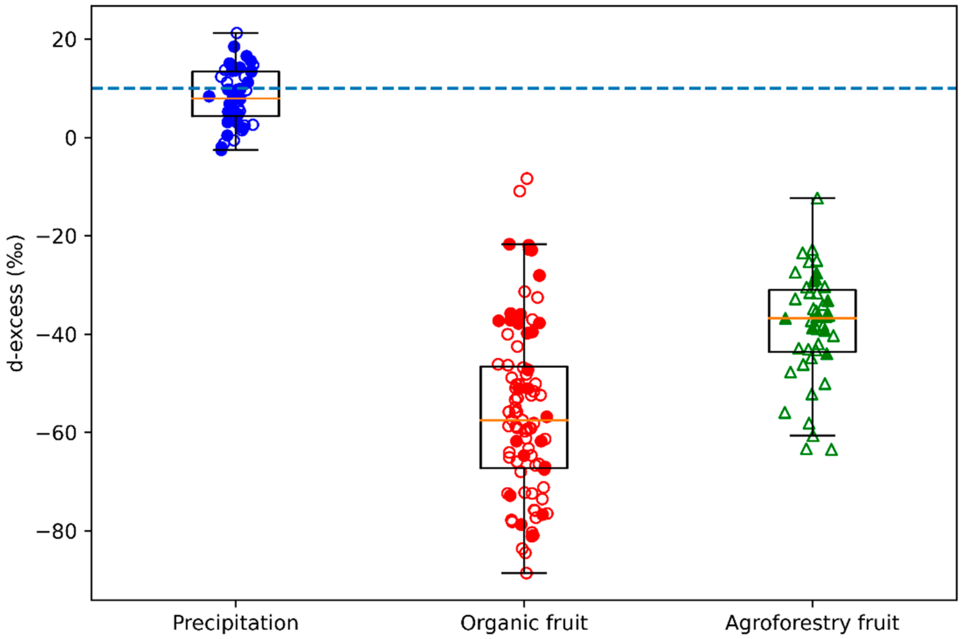

Figure S4. d-excess values ($d = \delta^2H - 8\cdot\delta^{18}O$) for precipitation in the Vlădești/Vâlcea region and raspberry fruit water. Filled symbols correspond to 2024 samples and open symbols to 2025 samples. The dashed horizontal line marks d-excess = 10‰ (intercept of the Global Meteoric Water Line). Boxes show medians and interquartile ranges; points represent individual samples.
